# Safety, tolerability, and efficacy of monoclonal CD38 antibody felzartamab in late antibody-mediated renal allograft rejection: study protocol for a phase 2 trial

**DOI:** 10.1186/s13063-022-06198-9

**Published:** 2022-04-08

**Authors:** Katharina A. Mayer, Klemens Budde, Philip F. Halloran, Konstantin Doberer, Lionel Rostaing, Farsad Eskandary, Anna Christamentl, Markus Wahrmann, Heinz Regele, Sabine Schranz, Sarah Ely, Christa Firbas, Christian Schörgenhofer, Alexander Kainz, Alexandre Loupy, Stefan Härtle, Rainer Boxhammer, Bernd Jilma, Georg A. Böhmig

**Affiliations:** 1 Division of Nephrology and Dialysis, Department of Medicine III, Medical University of Vienna, Währinger Gürtel 18-20, A-1090 Vienna, Austria; 2 Department of Nephrology, Charité University Medicine Berlin, Berlin, Germany; 3 Alberta Transplant Applied Genomics Centre, Faculty of Medicine & Dentistry, University of Alberta, Edmonton, Alberta Canada; 4 Nephrology, Hemodialysis, Apheresis and Kidney Transplantation Department, University Hospital Grenoble, Grenoble, France; 5 Department of Clinical Pathology, Medical University of Vienna, Währinger Gürtel 18-20, A-1090 Vienna, Austria; 6 Department of Clinical Pharmacology, Medical University of Vienna, Vienna, Austria; 7 INSERM UMR 970, Paris Translational Research Centre for Organ Transplantation, Université de Paris, Paris, France; 8 MorphoSys AG, Planegg, Germany

**Keywords:** Antibody-mediated rejection, CD38, Donor-specific antibody, Felzartamab, Kidney transplantation, Monoclonal antibody, Natural killer cell, Plasma cell

## Abstract

**Background:**

Antibody-mediated rejection (ABMR) is a cardinal cause of renal allograft loss. This rejection type, which may occur at any time after transplantation, commonly presents as a continuum of microvascular inflammation (MVI) culminating in chronic tissue injury. While the clinical relevance of ABMR is well recognized, its treatment, particularly a long time after transplantation, has remained a big challenge. A promising strategy to counteract ABMR may be the use of CD38-directed treatment to deplete alloantibody-producing plasma cells (PC) and natural killer (NK) cells.

**Methods:**

This investigator-initiated trial is planned as a randomized, placebo-controlled, double-blind, parallel-group, multi-center phase 2 trial designed to assess the safety and tolerability (primary endpoint), pharmacokinetics, immunogenicity, and efficacy of the fully human CD38 monoclonal antibody felzartamab (MOR202) in late ABMR. The trial will include 20 anti-HLA donor-specific antibody (DSA)-positive renal allograft recipients diagnosed with active or chronic active ABMR ≥ 180 days post-transplantation. Subjects will be randomized 1:1 to receive felzartamab (16 mg/kg per infusion) or placebo for a period of 6 months (intravenous administration on day 0, and after 1, 2, 3, 4, 8, 12, 16, and 20 weeks). Two follow-up allograft biopsies will be performed at weeks 24 and 52. Secondary endpoints (preliminary assessment) will include morphologic and molecular rejection activity in renal biopsies, immunologic biomarkers in the blood and urine, and surrogate parameters predicting the progression to allograft failure (slope of renal function; iBOX prediction score).

**Discussion:**

Based on the hypothesis that felzartamab is able to halt the progression of ABMR via targeting antibody-producing PC and NK cells, we believe that our trial could potentially provide the first proof of concept of a new treatment in ABMR based on a prospective randomized clinical trial.

**Trial registration:**

EU Clinical Trials Register (EudraCT) 2021-000545-40. Registered on 23 June 2021. ClinicalTrials.gov NCT05021484. Registered on 25 August 2021

**Supplementary Information:**

The online version contains supplementary material available at 10.1186/s13063-022-06198-9.

## Background

Antibody-mediated rejection (ABMR) is a dominant cause of kidney allograft failure [1–3]. This type of rejection, commonly triggered by preformed or de novo anti-human leukocyte antigen (HLA) donor-specific antibodies (DSA), is a prevalent finding in late indication biopsies. Its diagnosis, which is based on distinct serological, morphologic, and molecular criteria [4], is associated with a progressive decline in renal function [2]. While continuous diagnostic refinement has helped define the role of this rejection type as a major trigger of chronic transplant injury, treatment of late ABMR still represents a major challenge [5, 6]. Recent randomized controlled trials have failed to demonstrate the efficacy of several widely used therapeutic approaches, such as proteasome inhibition (bortezomib) [7], CD20 antibody rituximab plus high-dose intravenous immunoglobulin (IVIG) [8], or terminal complement blockade using the anti-C5 monoclonal antibody eculizumab [9]. Over the last few years, interference with the interleukin-6 (IL-6)/interleukin-6 receptor (IL-6R) axis to modulate the activation and development of B cells and antibody production has increasingly become of interest [10, 11], but the results of an ongoing large pivotal phase 3 trial to evaluate the safety and efficacy of anti-IL-6 antibody clazakizumab in chronic ABMR are still pending (IMAGINE; ClinicalTrials.gov identifier: NCT03744910) [12].

One promising immunotherapeutic target may be CD38, a 43.7-kDa type II transmembrane protein primarily expressed on immune and hematopoietic cells, with particularly high expression levels on antibody-producing plasma cells (PC) and natural killer (NK) cells [13]. CD38 exhibits ecto-enzymatic activity as nicotinamide-adenine dinucleotide-glycohydrolase/adenosine diphosphate-ribosyl cyclase and may play a role as an adhesion molecule (interaction with CD31) and cell-activating receptor that upon ligation triggers proliferation and cytokine production [13]. Monoclonal antibodies against CD38 are known to be highly effective in the treatment of multiple myeloma [14]. The mechanisms of action include depletion of malignant PC via complement-dependent cytotoxicity, antibody-dependent cellular cytotoxicity/phagocytosis, and/or apoptotic signaling [15–19].

We speculate that CD38 antibody treatment may also effectively deplete DSA-producing PC and thus, by reducing the load of deleterious alloreactivity, counteract tissue inflammation and injury in ongoing ABMR. In addition, one may speculate that targeting CD38 could also interfere also with a key pathomechanism in ABMR, namely NK cell-triggered tissue injury. Indeed, recent studies have suggested a dominant role of NK cells, e.g., activated upon engagement of Fc gamma receptor IIIA (CD16) with endothelium-bound IgG [20, 21]. Moreover, there is even evidence for a role of NK cell activation via a “missing self” mechanism, which may act independently of DSA [22, 23]. In a recently published case of ABMR (associated with smoldering myeloma), a 9-month course of CD38 antibody daratumumab was reported to successfully reverse severe chronic active ABMR [24]. Treatment led to a marked reduction of DSA (associated with a depletion and modulation of bone marrow-derived alloantibody-producing PC) and, in parallel, a profound reduction in peripheral blood NK cell counts and intra-graft NK cell infiltrates [24]. Also, in a recent experimental study (sensitized rhesus macaques, transplant model), targeting CD38 was shown to significantly reduce DSA and to prolong renal allograft survival [25]. The same authors reported a significant decrease in DSA in two clinical cases (combined heart and kidney allograft recipient with refractory ABMR and a highly sensitized heart transplant candidate), leading to clinical improvement of rejection and accelerated heart graft access, respectively [25].

Here, we detail the protocol of a 12-month randomized placebo-controlled parallel-group trial, the first in an organ transplant setting, primarily designed to evaluate the safety and tolerability of felzartamab (MOR202; MorphoSys AG, Planegg, Germany) [26], in a cohort of 20 renal allograft recipients with late ABMR. Felzartamab is a recombinant fully human monoclonal CD38 antibody (IgG1) derived from a proprietary antibody phage library, initially developed for the treatment of multiple myeloma, and currently being evaluated in autoimmune disease (membranous nephropathy; ClinicalTrils.gov Identifier: NCT04145440; NCT04733040). The design of our trial, which will include two sequential follow-up protocol biopsies to evaluate the impact of targeting CD38 on microvascular inflammation (MVI), patterns of gene expression (Molecular Microscope® System; MMDx), and chronic transplant injury, will provide a preliminary efficacy assessment providing first data on effect size and variability. Our trial may provide a useful basis for the design of larger long-duration trials in this specific context.

## Methods/design

### Study design and trial flow

This multi-center study (University of Vienna, Austria; *Charité* Universitätsmedizin Berlin; University Hospital Grenoble, France) is planned as an investigator-driven randomized controlled, double-blind phase 2 trial designed to assess the safety, tolerability, pharmacokinetics (PK), immunogenicity, pharmacodynamics (PD), and efficacy (preliminary assessment) of the fully human CD38 monoclonal antibody felzartamab in kidney transplant recipients with late active or chronic-active ABMR. The sponsor of this non-commercial trial, the Medical University of Vienna, will carry out the trial (in collaboration with research partners) and is responsible for all associated scientific, ethical, regulatory, and legal aspects. The funder, MorphoSys AG (Planegg, Germany), has set funding conditions and will provide external funding.

We hypothesize that felzartamab will have an acceptable safety profile in kidney transplant recipients on baseline immunosuppression. Moreover, we hypothesize that repeated administration of felzartamab is able to counteract tissue inflammation and injury in ongoing ABMR, in particular, MVI, HLA antigen-specific B cell alloresponses, and, as a consequence, alloantibody/NK cell-triggered chronic graft injury.

**Fig. 1:**
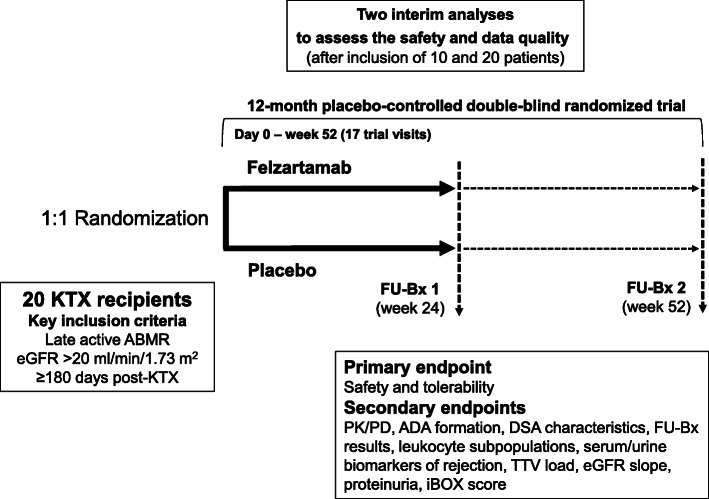
Study flowchart. ADA, anti-drug antibody; DSA, donor-specific antibody; eGFR, estimated glomerular filtration rate; EP; endpoint; FU-Bx, follow-up biopsy; KTX, kidney transplantation; PD, pharmacodynamics; PK, pharmacokinetics; TTV, torque teno virus

A flowchart for the study is provided in Figure 1. The trial will include 20 kidney transplant recipients with late ABMR. Participants will be randomized to receive felzartamab or placebo for a period of 6 months. The primary objective of the trial will be to assess the safety and tolerability of a 6-month course of treatment over a period of 12 months. Additionally, the trial will provide the first data of felzartamab in this indication on pharmacokinetics and pharmacodynamics (peripheral blood PC and NK cell depletion), efficacy (progression/activity of rejection in two sequential follow-up allograft biopsies, blood biomarkers), and surrogate parameters reflecting the clinical progression of allograft dysfunction (slope of eGFR [27] and a recently validated prediction system for risk of allograft loss (iBOX) [28]). Preliminary efficacy results may provide a valuable basis for the potential design of a pivotal trial powered for the detection of meaningful clinical outcome differences. We expect the recruitment phase to last 12–18 months and a total study duration of 24–30 months.

### Participants

We plan to include 20 kidney transplant recipients with anti-HLA DSA in serum and biopsy features of late (≥ 180 days post-transplant) active or chronic active ABMR in an indication biopsy (index biopsy; performed as part of the clinical routine for a positive post-transplant DSA result and slow deterioration of allograft function and/or proteinuria). Other key inclusion criteria are an age > 18 years, a functioning graft at ≥ 180 days post-transplantation, and an eGFR ≥ 20 ml/min/1.73 m^2^. A complete list of inclusion and exclusion criteria is provided in Table 1. Eligible patients will be identified upon routine monitoring of transplant recipients in outpatient care. Standardized protocols for surveillance and indication allograft biopsies will ensure timely diagnosis of ABMR and thus adequate patient enrolment to reach the target sample size. To promote participant retention and complete follow-up, all study participants will be asked to provide, at the beginning of the study, their updated contact information.

**Table 1:** Inclusion and exclusion criteria

**Inclusion criteria**	1. Voluntary written informed consent
	2. > 18 years (maximum 80 years)
	3. Functioning living or deceased donor allograft after ≥ 180 days post-transplantation
	4. eGFR ≥ 20 ml/min/1.73 m^2^ (CKD-EPI formula)
	5. HLA class I and/or II antigen-specific antibodies (preformed and/or de novo DSA)
	6. Active or chronic/active ABMR (± C4d in PTC) according to the Banff 2019 classification
	7. Molecular ABMR score (MMDx) ≥ 0.2
**Exclusion criteria**	1. Patients actively participating in another clinical trial
	2. Age ≤ 18 years
	3. Female subject is pregnant or lactating or not on adequate contraceptive therapy
	4. ABO-incompatible transplant
	5. Index biopsy results:
	a. T cell-mediated rejection classified Banff grade ≥ I
	b. De novo or recurrent severe thrombotic microangiopathy
	c. Polyoma virus nephropathy
	d. De novo or recurrent glomerulonephritis
	6. Acute rejection treatment ≤ 3 months before screening 7. Previous treatment with other CD38 monoclonal antibodies (e.g., daratumumab) 8. Previous treatment with other immunomodulatory monoclonal/polyclonal antibodies (e.g., CD20 Ab rituximab, IL-6/IL-6R Ab) ≤ 3 months before study treatment 9. Total bilirubin > 2 × the upper limit of normal [ULN], alanine transaminase, and aspartate aminotransferase > 2.5 × ULN 10. Hemoglobin < 8 g/dL 11. Thrombocytopenia: platelets < 100 × 10^9^/L 12. Leukopenia: leukocytes < 3 × 10^9^/L 13. Neutropenia: neutrophils < 1.5 × 10^9^/L 14. Hypogammaglobulinemia: serum IgG < 400 mg/dL
	15. Active viral, bacterial, or fungal infection precluding intensified immunosuppression
	16. Active malignant disease precluding intensified immunosuppressive therapy
	17. Latent or active tuberculosis (positive QuantiFERON-TB-Gold test)
	18. Administration of a live vaccine within 6 weeks of screening
	19. History of alcohol or illicit substance abuse
	20. Serious medical or psychiatric illness likely to interfere with participation in the study

*ABMR* antibody-mediated rejection, *ALT* alanine aminotransferase, *AST* aspartate aminotransferase, *DSA* donor-specific antibody, *eGFR* estimated glomerular filtration rate, *CKD-EPI* Chronic Kidney Disease Epidemiology Collaboration, *HLA* human leukocyte antigen, *IL-6* interleukin-6, *IL-6* IL-6 receptor, *MMDx* Molecular Microscope® Diagnostic platform, *ULN* upper limit of normal

### Randomization procedure

After patients have given informed consent (Additional file 1) and completed the screening phase subjects eligible for study inclusion will be randomized on the day of study initiation. Randomization will be performed by computer assignment using a web-based randomization platform (www.meduniwien.ac.at/randomizer) in a 1:1 ratio to one of the two study arms (felzartamab versus placebo). Randomization will be stratified by study site and according to the ABMR categories (active versus chronic/active ABMR) to ensure a balance of patients with these two histological types between the two arms. For each patient, a study ID will be assigned. Individual roles of investigators, study nurses, and clinical pharmacists will be defined within the online randomization tool.

### Sample size calculation

As the effect size for this study is unknown (there is no sufficient prior information to base a sample size on), it is not possible to perform a sample size estimation using an efficacy test. An important aspect of this trial is to evaluate whether there are any safety issues. A preliminary assessment of efficacy outcomes will provide the first systematic results (including the extent of variation) on the effect of felzartamab on important molecular, morphological, immunological, and clinical endpoints (measurement of the capacity of beneficial change).

### Interventions

The study medication will be administered only by authorized blinded study personnel. Every effort will be made to ensure timely administration as defined in the protocol. If medication cannot be administered, the reason has to be documented in the case report form.

#### Felzartamab/placebo

Patients will be dosed with felzartamab or placebo for a period of 6 months. Both felzartamab (16 mg/kg per infusion) and placebo will be administered as an intravenous infusion. Felzartamab (supplied and provided by MorphoSys AG, Planegg, Germany) will be diluted with 250 mL 0.9% sodium chloride solution prior to administration. The placebo medication will be administered with normal saline for infusion and will be provided by the investigator. As transplant patients are on multi-compound immunosuppressive baseline therapy, and therefore at increased risk of infections, we plan a reduction in dosing intervals after the first cycle. Nine doses of felzartamab will be administered as intravenous infusions over 6 treatment cycles. Dosing occurs every week in cycle 1 (C1) and every 4 weeks in cycles 2–6. The first two infusions will be administered slowly (over approximately 90 min), and, if no infusion reactions occur, infusion times may be shortened to 1 h or shorter (minimum 30 min) for subsequent infusions. If infusion reactions occur, the infusion may be halted temporarily, or stopped in its entirety, depending on the severity of the reaction. In some cases, premedication may be extended to subsequent infusions. The infusion rate for resuming application will be slower.

#### Premedication

To prevent infusion-related reactions, patients allocated to the felzartamab arm will receive intravenous premedication prior to the first two infusions (day 0 and day 14). Patients in the placebo arm will receive placebo (0.9% NaCl solution). Premedication will be administered 30 min before the infusion of felzartamab and will consist of diphenhydramine (30 mg), paracetamol (1000 mg), and prednisolone (100 mg) (each in 100 mL volume). In the placebo arm, patients will receive 3 × 100 mL NaCl 0.9%.

#### Prohibited medication/treatments

Prohibited medication/treatments include rituximab, eculizumab, proteasome inhibitors, IVIG, plasma exchange or immunoadsorption, other investigational drugs/treatments including commercially available CD38, or anti-IL-6R monoclonal antibody drugs such as daratumumab or tocilizumab. Given the fact that there is currently no treatment that has been proven effective in late ABMR [5, 6], withholding any of the above-listed treatments is not expected to harm the participants.

### Baseline immunosuppression

All recipients on a calcineurin inhibitor (tacrolimus or cyclosporine A [CyA]) or a mammalian target of rapamycin (mTOR) inhibitor without azathioprine or mycophenolic acid (MPA) will receive mycophenolate mofetil or enteric-coated mycophenolic acid (EC-MPA) to avoid under-immunosuppression [initially at a dose of 2 × 500 mg (or 2 × 360 mg, respectively) per day; stepwise increase to 2 × 1000 mg (or 2 × 720 mg) per day if tolerated]. For patients developing hematologic toxicity, a limited sampling strategy to estimate the MPA area under the curve and/or assessment of inosine monophosphate dehydrogenase activity may be considered to detect MPA overexposure [29, 30]. Tacrolimus will be adjusted to achieve target trough levels between 5 and 10 ng/mL, CyA to 80–120 ng/mL, sirolimus to 5-10 ng/mL, and everolimus to 3–8 ng/mL. CNI and mTOR trough levels (and thus also medication adherence) will be monitored at regular intervals. Recipients weaned off steroids will receive low-dose prednisolone (5 mg/day).

### Blinding and unblinding

In order to minimize bias, the study is designed as a double-blinded trial. MorphoSys AG will provide the investigational medical product (felzartamab) to a contract manufacturing organization located in Vienna, Austria (ABF Pharmaceutical Services GmbH), for storage, labeling, and shipment to the participating study sites. Randomization will be conducted at each study site by non-blinded pharmacists (with the treating team remaining blinded) responsible for the preparation of premedication and felzartamab as well as placebo (infusions of normal saline). The investigational drug and placebo will be formulated to be identical in color, appearance, and smell. The participating investigators, including those assessing the outcomes, medical staff interacting with patients, and study participants, will be unaware of the randomization sequence. They will be unable to access the secure allocation list (restricted password-protected access) until the end of the trial. The randomization sequence will be unblinded by a dedicated clinical pharmacist (according to his/her role defined in the randomization tool) after the last patient has completed the trial. Premature unblinding (treatment code envelopes will be securely stored on site) may be necessary in cases of medical emergencies or serious medical conditions, where participants cannot be treated adequately unless the medical staff knows the allocated treatment or reports of suspected unexpected serious adverse events. Unblinding can, if necessary, be requested by the data and safety monitoring board (DSMB).

### Outcome parameters

**Fig. 2:**
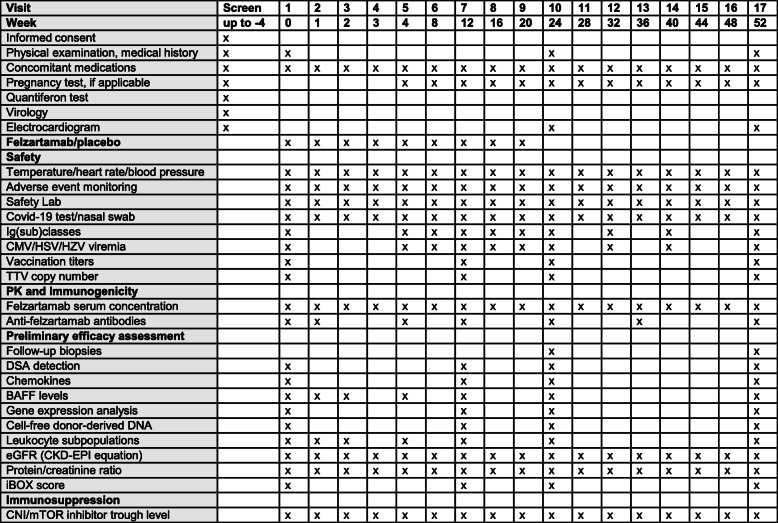
Schedule of events. CMV, cytomegalovirus; CNI, calcineurin inhibitor; DSA, donor-specific antibody; eGFR, estimated glomerular filtration rate; HSV, herpes simplex virus; HZV, herpes zoster virus; Ig, immunoglobulin; mTOR, mammalian target of rapamycin; PK, pharmacokinetics; TTV, torque teno virus

Primary and secondary outcome measures are detailed in Table 2. A schedule of events is provided in Figure 2. Primary outcome measures are the safety and tolerability of felzartamab, evaluated throughout the study period (17 visits until the end-of-study visit at 52 weeks: day 0, week 1, 2, 3, 4, 8, 12, 16, 20, 24, 28, 32, 36, 40, 44, 48, and 52). According to the International Conference on Harmonization (ICH) statistical principles, safety concerns the medical risk to the subjects, as assessed by laboratory tests, vital signs, and adverse events, while tolerability describes the degree to which overt adverse effects can be tolerated by the subject (as reflected by the rate of dropouts due to a lack of tolerability). Major secondary endpoints include the course of DSA (and in parallel total Ig and IgG subclass levels); the dynamics of peripheral blood counts of PC, NK cells, and T and B cell subpopulations; and biomarkers of rejection, B cell immunity, and overall immunosuppression. Moreover, 6- and 12-month renal allograft biopsies will be assessed for morphological and molecular rejection criteria. Clinical endpoints will include validated surrogate endpoints to predict long-term allograft survival.

**Table 2:** Trial endpoints

**Primary outcome**	Safety and tolerability (every visit)
**Secondary outcomes**	**Every visit**
	PK of felzartamab (antibody levels)
	Kidney function (eGFR)
	Urinary protein excretion (protein/creatinine ratio)
	Biopsy-proven acute rejection necessitating rejection treatment
	Graft loss, death
	**Day 0 and weeks 24 and 52 (additional visits as indicated in the protocol)**
	Anti-felzartamab antibodies
	Chemokines, BAFF
	Effect on leukocyte subsets in peripheral blood
	Cell-free donor-derived DNA
	Effect on gene expression in peripheral blood cells
	DSA characteristics
	DSA-MFI
	Number of DSA
	Ig classes (IgG, IgA, IgM) and IgG subclasses (IgG1, 2, 3, 4)
	TTV load
	iBOX score
	**Week 24 and week 52**
	Protocol biopsy results
	ABMR category
	Microcirculation inflammation (glomerulitis, peritubular capillaritis)
	Transplant glomerulopathy and interstitial fibrosis/tubular atrophy
	Molecular ABMR score
	Archetype analysis of gene expression profiles

*DSA* donor-specific antibody, *eGFR* estimated glomerular filtration rate, *HLA* human leukocyte antigen, *Ig* immunoglobulin, *MFI* mean fluorescence intensity, *PD* pharmacodynamics, *PK* pharmacokinetics, *TTV* torque teno virus

### Safety evaluation and reporting of adverse events

There are currently no systematic data available on the safety and tolerability of felzartamab in transplant patients. From a previous 1–2a trial, however, safety data are available for a cohort of 91 patients with refractory or relapsing MM. In this study, felzartamab was applied as the sole treatment or in combination with dexamethasone or immunomodulatory compounds, and its use was considered safe for the intended population [26].

Within the current phase 2 trial, predefined, predefined safety assessments include regular pregnancy tests in women of childbearing potential, safety lab and immunosuppressive drug monitoring at every study visit, assessment of vital signs (heart rate, blood pressure, body temperature, in case of clinical symptoms monitoring of SpO_2_ and respiratory rate before and in 30 min intervals during and after infusion of the antibody), IgG levels to detect hypogammaglobulinemia, regular COVID-19 antigen or PCR testing, monitoring for cytomegalovirus (CMV), herpes simplex (HSV) and herpres zoster (HZV) viremia, and indication allograft biopsies in case of deterioration of graft function. In any clinical situation, including emergencies, adequate medical care will be provided. Safety evaluation will include careful monitoring of all adverse events (AE), including serious adverse events (SAE) defined according to the International Conference on Harmonization (ICH) guidelines and WHO GCP guidelines, suspected unexpected serious adverse reactions (SUSAR), and adverse events of special interest. AE will be classified using the Medical Directory for Regulatory Activities (MedDRA), and their severity will be graded on a 3-point scale (mild, moderate, severe). The causal relationship between the study drug and the AE will be documented according to the investigator’s clinical expertise and judgment. SUSAR will be reported to the institutional ethics committees and competent authorities. A Development Safety Update Report (DSUR) will be submitted annually.

### Interim analyses

The trial will be monitored by a DSMB to assess the safety and data quality. The DSMB is independent of the sponsor and competing interests. To detect any difference in safety issues noted between the groups in a timely fashion, the board members will be instructed to perform interim analyses after 10 and 20 patients have been randomized and have started the trial (depending on the duration of the recruitment period, for interim analyses, a complete patient follow-up may not be available). The DSMB will analyze the recorded AE and safety lab results in relation to the randomization sequence and may consider stopping the trial if the overall pattern of related SAE or changes in safety lab results strongly support a major safety signal. The exact statistical definitions of criteria for premature study termination are not defined.

### Criteria for patient withdrawal and premature termination of the trial

Study participants may prematurely discontinue from the study at any time. Study participation will be terminated for any of the following reasons: withdrawal of informed consent; any AE, laboratory abnormality, or intercurrent illness which, in the opinion of the investigator, indicates that continued participation in the study is not in the best interest of the subject; new onset of pregnancy; termination of the study by the sponsor; loss of ability to freely provide consent through imprisonment or involuntary incarceration for treatment of either a psychiatric or physical illness; inability to comply with the protocol; by the discretion of the investigator; evidence of confirmed hepatic decompensation (Child-Pugh class B or C, score > 6); alanine aminotransferase (ALT) ≥ 5× baseline or ≥ 10× upper limit of normal (ULN) and either total bilirubin > 2× ULN or international normalized ratio (INR) > 2× ULN; platelet count < 25× 10^9^/L; and any grade 4 AE or clinical laboratory finding considered to be related to the study drug. In case of premature discontinuation after study drug intake, the investigations scheduled for the end-of-study visit will be performed 28 days after study drug discontinuation. The subjects will be advised that participation in these investigations is voluntary. Furthermore, they may request that from the time point of withdrawal no more data will be recorded and that all biological samples collected in the course of the study will be destroyed.

The sponsor has the right to close this study at any time. The trial or single-dose steps will be terminated prematurely (i) if the overall pattern of related SAE or changes in safety lab results strongly support a major safety signal and (ii) if the number of dropouts is so high that proper completion of the trial cannot realistically be expected.

### Post-trial care

Specific post-trial care will not be provided. Included patients are insured against any harm arising from the study interventions.

### Data management and confidentiality

Designated investigator staff will enter the data required by the protocol into the study case report form (CRF). Investigators will certify that the data entered is complete and accurate. All study-related information will be stored securely at the study site, in areas with limited access, and only study personnel will have access to these data. To maintain participant confidentiality, laboratory specimens, reports, data collection, and administrative forms will be identified by a coded identification number only. Records containing names or other personal identifiers, including informed consent forms, will be stored separately from study records identified by code number.

### Quality control and quality assurance

The designated monitor will contact and visit the investigator regularly and will be allowed to have access to all source documents needed to verify the entries in the CRF and other protocol-related documents. The monitor will work according to a monitoring plan and will provide a report after each visit. It will be the monitor’s responsibility to verify adherence to the protocol and the completeness, consistency, and accuracy of entered data (verification for the presence of informed consent, adherence to the inclusion/exclusion criteria, documentation of AE, and the recording of the main efficacy, safety, and tolerability endpoints). The investigator will resolve discrepancies in data. Upon request, the investigator will make all study-related source data and records available to a qualified quality assurance auditor mandated by the sponsor or to competent authority inspectors. The main purposes of an audit or inspection are to confirm that the rights and welfare of the subjects have been adequately protected and that all data relevant for the assessment of safety and efficacy of the investigational product have appropriately been reported to the sponsor in accordance with applicable legislation.

### Protocol modifications

Important protocol changes as well as changes in the eligibility criteria, outcomes, or analyses will be communicated to the investigators, institutional ethics committee, regulatory authorities, trial participants, and trial registries, respectively.

### COVID-19

The study team will adhere to all COVID-19 measures in place and will ensure that no resources required to combat the pandemic are being used for study purposes. They will make sure that sufficient personnel are available and that the participants are not exposed to any additional risk of infection through their participation in the study. COVID-19 antigen or PCR tests will be carried out at every visit (or before administration of study medication/placebo). In case of a positive test result, the study medication will not be administered, and patient care will follow the current recommendations and guidelines.

### Methodology

#### Biobanking

As defined in the study protocol, biological specimens (serum, plasma, peripheral blood cells, RNA isolated from biopsies) will be collected and biobanked for retrospective evaluation, including pharmacokinetic measurements, HLA antibody and chemokine/cytokine detection, flow cytometry, detection of donor-derived cell-free DNA, and gene expression analysis. Patients will provide consent for sample collection for secondary endpoint analysis within the planned trial. Aliquots will be frozen at − 80 °C and stored in specifically designated refrigerators. The samples will be anonymized so that no conclusions can be drawn about individual patients in the involved laboratories.

#### Kidney function

eGFR will be assessed using the Chronic Kidney Disease Epidemiology Collaboration (CKD-EPI) equation (mL/min/1.73m^2^). Protein excretion will be documented as protein/creatinine ratio in spot urine (mg/g).

#### Pharmacokinetics and immunogenicity

For the determination of felzartamab serum concentrations (PK) and the potential development of anti-falzartamab antibodies, serum samples will be collected throughout the study. The assessment will be performed with drug-specific validated ligand binding assays.

#### Transplant biopsies

Follow-up biopsies will be performed at weeks 24 and 52 (end-of-study visit), after exclusion of a coagulation disorder or a platelet count below 80%. Anticoagulants or inhibitors of thrombocyte aggregation will be paused prior to biopsy. The biopsy will be performed under local anesthesia (lidocaine) using ultrasound-guided percutaneous techniques (1–2 cores per biopsy, 16 gauge needle). After the biopsy has been performed, patients will be monitored for complications (serial blood pressure measurements, monitoring for hematuria, hemoglobin check 4 h after biopsy) for 5 to 8 h. Histomorphology will be evaluated on paraffin-embedded sections using standard methodology. For immunohistochemical complement C4d staining, we will use a polyclonal anti-C4d antibody (BI-RC4D, Biomedica; Vienna, Austria); minimal immunohistochemical staining (C4d Banff score ≥ 1) along peritubular capillaries will be considered positive. Biopsies will also be evaluated by electron microscopy for the detection of multilayering of peritubular capillary basement membranes (MLPTC). Morphological results will be read locally (Medical University of Vienna, Charité Universitätsmedizin Berlin) in a blinded fashion. At the end of the trial, biopsies will be scanned and re-evaluated by a central pathologist (Heinz Regele, Medical University of Vienna). In addition, all biopsies will also be analyzed using the internationally validated Molecular Microscope® Diagnostic System (MMDx) platform [31, 32]. For each biopsy, a 3-mm portion of one core will be placed in RNAlater immediately, stored at − 20 °C and shipped to the Alberta Transplant Applied Genomics Centre (ATAGC, University of Alberta, Edmonton, AB, Canada) at ambient temperature. There, gene array analysis will be performed. Thoroughly validated molecular scores based on machine-learning derived lesion-based classifiers related to rejection [ABMR, T cell-mediated rejection (TCMR), all rejection], inflammation (global disturbance score), or chronic injury (atrophy/fibrosis score) will be generated using a reference set of > 1500 biopsies. Moreover, gene expression patterns will be evaluated using unbiased archetype analysis [32]. For the classification of ABMR according to the Banff 2019 scheme, all biopsy results will be analyzed in the context of the molecular results. ABMR categories and morphological single lesions will be defined and scored following the 2019 update of the Banff classification [4]. ABMR will be defined on the basis of both morphological (histomorphology, immunohistochemistry, electron microscopy) and thoroughly validated molecular criteria: (i) evidence of acute or chronic tissue injury, (ii) evidence of current/recent antibody interaction with the vascular endothelium, and (iii) serological evidence of DSA.

#### HLA antibody detection

For the assessment of the course of HLA antibody levels, serum samples will be evaluated after completion of the study according to published protocols [7, 11]. For antibody detection, LABscreen single-antigen flow-bead assays (One Lambda, Canoga Park, CA) will be applied. Serum samples will be incubated with ethylenediaminetetraacetic acid (EDTA, 10 mM) to prevent complement interference [33]. Data acquisition will be performed via a LABScan™ 200 flow analyzer (Luminex Corporation, Austin, TX, USA). For longitudinal analysis of DSA/HLA antibody levels, bead assays will be performed retrospectively (centralized analysis) to avoid influences of day-by-day variations on test results (test batches will include samples from 4 to 6 patients each). Donor specificity will be defined according to serological and/or low- or high-resolution donor/recipient HLA typing (HLA-A, HLA-B, HLA-Cw, HLA-DR, HLA-DQ, HLA-DP upon availability) provided either by the local HLA lab or the Eurotransplant database. Test results will be documented as the mean fluorescence intensity (MFI) of the immunodominant DSA. An MFI threshold > 1000 will be considered positive. To estimate the impact of felzartamab treatment on DSA levels, we will document the percent change in MFI. In an effort to quantify the changes in DSA levels more accurately, we plan to additionally perform dilution experiments following a protocol described previously [11]. Briefly, non-linear standard curves based on raw DSA MFI levels (immunodominant DSA) will be obtained by serial dilution of individual patient sera collected prior to the start of treatment (all samples will be incubated with EDTA) and at week 24. According to computed standard curves, the fold change of antibody levels will then be calculated from DSA MFI levels detected in the same experiment for undiluted week 12, week 24, and week 52 samples.

#### Immunoglobulin (Ig) levels

Total IgG, IgM, IgA, and IgG subclasses will be assessed in the serum applying immunonephelometry on a BN™ II analyzer (Siemens Healthineers, Erlangen, Germany).

#### Immunologic biomarkers

For chemokine detection, we will use a Luminex-based protocol as described earlier [34]. For quantification of *chemokine (C-X-C motif) ligand (CXCL)9 and CXCL10*, serum samples will be adjusted to 10 mM EDTA to prevent complement interference. Undiluted samples will be measured in duplicates using multiplexed Human ProcartaPlex Simplex Immunoassays (Thermo Fisher Scientific, Waltham, MA, USA) according to the manufacturer’s instructions. Immunoassays will be performed on a Luminex 200 instrument (Luminex Corp., Austin, Tx, USA). Urinary results will be normalized to creatinine excretion and presented as pg (chemokine)/mg (creatinine). Levels of donor-derived cell-free DNA in recipient plasma samples reflecting the extent of ongoing allograft injury will be detected using standard technology, based on the detection of a defined set of single nucleotide polymorphisms detected by next-generation sequencing on an Illumina MiSeq sequencer (Illumina Inc., San Diego, CA, USA) [12]. BAFF levels will be detected using Luminex or enzyme-linked immunosorbent assay (ELISA) technology using commercial kits

#### Leukocyte subpopulations

The underlying mechanisms of chronic antibody-mediated rejection, especially the role of peripheral T and B cell subsets have not yet been fully clarified. Thus, the prospective monitoring of immune phenotype under therapy with felzartamab is a promising approach to further elucidate the impact on immune-regulatory pathways when CD38 is targeted. Moreover, assessment of PC and NK cell counts allows for the monitoring of the pharamacodynamic effects of the CD38 antibody. For the monitoring of leukocyte (sub) populations, we will use reproducible immune monitoring panels for phenotyping. Recently, the international “The ONE study” consortium has designed a standardized panel (DuraClone®) for flow cytometry-based immune phenotyping that demonstrated robust results [35]. In the DuraClone kits, pre-defined assay tubes contain a layer with a dried-down antibody panel ready to use. Up to 10 different monoclonal antibodies per tube allow the identification of leukocyte (e.g., T cell, B cell, NK cell subsets) subpopulations present in whole blood samples. For flow cytometric assessment of CD38 expression, assays will be modified to include staining with non-crossreactive CD38 monoclonal antibodies.

#### Gene expression analysis

For gene expression analysis, 5 mL of blood will be collected in PAXgene Blood RNA tubes and stored at − 80 °C until the retrospective analysis is performed. These tubes are designed for the stabilization of RNA in the blood during long-term storage at ultra-low temperatures. Gene expression pattern analyses (microarray analysis) will be performed from peripheral blood to evaluate the impact of felzartamab on antibody-producing cells, analyzing genes annotated as part of the B cell receptorsignaling pathway.

#### Torque teno virus (TTV) quantification

For TTV analysis, DNA will be extracted from plasma using the NucliSENS easyMAG platform (bioMeriéux, France) and eluted in 50 μL of elution buffer. TTV DNA will be quantitated by TaqMan real-time polymerase chain reaction (PCR), according to previously described protocols [36]. The quantitative PCR reactions will be performed in a volume of 25 μL using 2 × TaqMan Universal PCR Master Mix, containing 5 μL of extracted DNA, 400 nM of each primer, and 80 nM of the probe. Thermal cycling will be started for 3 min at 50 °C, followed by 10 min at 95 °C, and then by 45 cycles at 95 °C for 15 s, at 55 °C for 30 s, and at 72 °C for 30 s, using the CFX96 Real-time System (Bio-Rad, Hercules, CA). The results will be recorded as copies/mL.

#### Course of vaccination titers

Serum IgG titers specific for mumps, measles, and rubella (MMR) will be analyzed by the ELISA technique. COVID-19 antibody titers will be detected using commercial kits.

### Statistical methods

Analyses will be conducted according to the intention-to-treat principle. For qualitative variables (e.g., the occurrence of AE in the two treatment groups), absolute and relative frequencies will be calculated per treatment group. Data will be visualized via bar plots. Such nominal data will be compared using Fisher’s exact test for descriptive purposes. Transplant and patient survival or AE (SAE)-free survival will be evaluated using Kaplan-Meier analysis, and the log rank test will be applied for group comparisons. For quantitative data (e.g., group comparisons for DSA levels, glomerulitis (g) plus peritubular capillaritis (ptc) score, interstitial fibrosis/tubular atrophy (IF/TA) score, molecular rejection scores, eGFR, and protein/creatinine ratio), the number of valid observations, mean, standard deviation, standard error, median, minimum, and maximum will be calculated separately for each treatment group and each time point. Data will be visualized via boxplots and histograms. For descriptive purposes, only such continuous data will be analyzed using parametric or non-parametric (independent/dependent data) tests. These include the Mann-Whitney *U* test or the *t*-test as appropriate. For paired data (e.g., difference in g+ptc or IF/TA score between month 0 and month 12), paired *t* test or Wilcoxon test will be used as appropriate. Analysis of pharmacokinetics of felzartamab will include a description of the time evolution of antibody/drug concentration. The elimination half-life, maximum plasma concentration, time to reach maximum plasma concentration, clearance, and volume of distribution will be computed using standard software. GFR trajectories will be analyzed using a linear mixed model with eGFR values from 0 to 52 weeks (weeks 0, 4, 8, 12, 16, 20, 24, 28, 32, 36, 40, 44, 48, 52) as the dependent variable. Time and treatment as well as their interaction will be used as fixed effects. Furthermore, patient-specific random effects for intercept and slope will be specified. If *p*-values are provided, this is done for exploratory purposes only. Therefore, only unadjusted *p*-values are presented, and no correction for multiplicity will be applied. Multiple imputation will be used to handle missing data. For statistical analysis, IBM SPSS Statistics version 24 (IBM Corporation, Armonk, NY, USA) and SAS version 9.4. (the SAS Institute Inc., Cary, NC, USA) will be used.

### Study registration

The study has been approved by the Austrian regulatory authority (Federal Office for Safety in Health Care, Austrian Agency for Health and Food Safety) and was registered in the European Clinical Trials Database (EudraCT number: 2021-000545-40; prospective registration) and in a public clinical trial database (ClinicalTrials.gov NCT05021484; prospective registration).

## Discussion

For late (chronic) ABMR, no evidence-based treatment is available [5]. A recently published expert recommendation has been to optimize baseline immunosuppression, but plasmapheresis, IVIG, and/or rituximab—common “standard-of-care” in early active ABMR—were not recommended [6]. Indeed, a recent randomized controlled trial has failed to demonstrate any impact of rituximab plus high-dose IVIG on the course of chronic ABMR [8]. Similarly, bortezomib has been found to be ineffective in a placebo-controlled trial powered to detect eGFR slope differences [7]. Finally, a small pilot study revealed no meaningful effect of terminal complement blockade on ABMR activity, with an at best marginal effect on renal function [9]. Such disappointing results reinforce the need for innovative concepts for the treatment of late (chronic) ABMR [12].

While case reports and observational studies may help to identify innovative treatment concepts for clinical trials, systematic prospective trials are necessary to prove treatment efficiency. One useful approach may thus be the design of an initial short-duration trial including a limited number of subjects. The primary aim would be to evaluate the safety and tolerability of a new immunomodulatory treatment in an at-risk patient population, namely transplant recipients on multi-compound immunosuppressive treatment. At the same time, preliminary secondary efficacy outcomes would provide first information regarding the effect size and variability (e.g., of surrogate parameters), supporting the design of a subsequent large long-duration trial (e.g., phase 3). In the absence of any evidence-based treatment, the inclusion of a control group subjected to a prolonged period of placebo treatment may be ethically justifiable. A prominent example of a systematic evaluation of a new treatment principle in late (chronic) ABMR is current research efforts on the establishment of IL-6 antagonism as a new anti-rejection treatment [12]. In initial observational studies, the anti-IL-6R antibody tocilizumab appears to halt the progression of ABMR and stabilize renal function [10]. A subsequent study, once again uncontrolled and observational, however, revealed contradictory results [37]. More recently, the efficacy of the concept (IL-6 antagonism using anti-IL-6 antibody clazakizumab) was strongly supported by the results of a randomized controlled pilot trial (20 patients) [11]. Although this trial was not designed to prove an effect on clinical endpoints, treatment with clazakizumab was found to be associated with a change in eGFR slope, reduction of C4d deposition, and decreased molecular ABMR activity in 12-month follow-up biopsies. At the same time, relevant safety signals were recorded, most importantly, the occurrence of gastrointestinal complications, which reinforced the need for careful patient selection and monitoring [11]. Now, a large carefully designed pivotal phase 3 study is underway, powered to detect a meaningful effect of a 5-year treatment course with clazakizumab on graft survival (IMAGINE; ClinicalTrials.gov identifier: NCT03744910).

There is now recent experimental and clinical evidence suggesting that interference with the surface molecule CD38 could be an effective strategy in ABMR prevention and treatment [12, 38]. Efficacy of such treatment was suggested by a recent case report: one case of successful ABMR treatment after ABO blood group-incompatible kidney transplantation [39], two patients with severe heart allograft rejection [25, 40], and a case of chronic active HLA DSA-triggered ABMR in a renal transplant recipient [24]. The latter case may be of particular interest for the planned trial, as detailed immunological monitoring (serial follow-up biopsies including molecular analysis of rejection activity, HLA antibody levels, PC and NK cell monitoring in the peripheral blood and tissue) was suggestive of effective downregulation of ABMR activity following 9-month treatment with CD38 antibody daratumumab. A major effect observed in this patient was a substantial reduction in DSA-MFI, a modulation of HLA antibody production by antibody-secreting cells isolated from the bone marrow, and a depletion of circulating plasma cells. In parallel, a profound reduction in immunoglobulin levels was noted. Sequential follow-up biopsies showed a marked reduction in molecular rejection-associated scores. At the same time, we observed depletion of NK cells in the peripheral blood and in the allograft. Given the presumed key role of NK cells in the process of rejection, NK cell depletion may have substantially contributed to ABMR reversal [24]. In a non-human primate model of recipient desensitization using daratumumab in combination with plerixafor, Kwun et al. [25] reported downregulation of HLA antibodies and prolonged renal allograft survival. The authors, however, noted ABMR recurrence and suggested modulation of regulatory T and B cell function, which might have favored rejection [25]. In our recently published case of chronic ABMR, a 3-month follow-up biopsy revealed the occurrence of dense interstitial infiltrates and severe focal tubulitis suggestive of TCMR [24]. In the absence of molecular gene expression patterns indicating TCMR, the significance of this finding was unclear, but such observations, which may point to altered immune regulation, may warrant careful consideration and reinforce the need for regular monitoring of renal function, and early indication biopsies in case of graft dysfunction. Subclinical rejection will be uncovered in follow-up biopsies scheduled for 6 and 12 months after trial inclusion.

Currently, no data obtained in transplant patients on dual or triple immunosuppression are available. Hence, safety assessment remains a primary objective of this trial. Preliminary safety data on the use of felzartamab (with or without an immunomodulatory drug and/or dexamethasone) are available from a clinical phase 1–2a trial, obtained in a cohort of 91 patients with refractory or relapsing multiple myeloma [26]. In this trial, an acceptable safety profile was reported. The most common grade 3 or higher treatment-emergent AE were hematologic and included lymphocytopenia (39.6%), neutropenia (36.3%), leukopenia (29.7%), thrombocytopenia (14.3%), and anemia (12.1%). Hematologic toxicity was more frequent among patients treated with felzartamab in combination with immunomodulatory therapies. The most common grade ≥ 3 non-hematologic treatment-emergent AE were hypertension, pneumonia, respiratory tract infection, and diarrhea. Infusion-related reactions to felzartamab were reported in 20% of the patients (grade ≥ 3, 1.1%), and thus at substantially lower rates than those reported for two other approved CD38 antibodies (daratumumab and isatuximab) following intravenous administration. Moreover, observed lower rates of such events under combined regimens with dexamethasone may point to a beneficial effect of steroid-based immunosuppression in transplant patients. Available results reinforce that during the first cycle of felzartamab, adequate pre-treatment including steroids is necessary to minimize the risk of infusion-related reactions [26].

In the felzartamab multiple myeloma trial, there was no major signal towards an increased infection rate [26]. Similarly, in large phase 3 trials evaluating CD38 antibody daratumumab, rates of serious infections either did not increase, or increased only slightly, under antibody treatment [41, 42]. Nevertheless, on top of baseline immunosuppression, CD38 blockade may generally offer the potential for increased infection rates. Hence, careful patient follow-up in our trial will include close monitoring for infectious complications (bacterial, viral, and fungal infections). Considering a potentially increased risk of viral infections, patients under felzartamab will be regularly monitored for cytomegalovirus, herpes simplex, and herpes zoster virus viremia including two interim analyses evaluated by the DSMB. Patients with an active infection (including a positive Quantiferon test) will be excluded from the trial. Moreover, patients will be monitored for IgG levels and substituted with IVIG in case of severe hypogammaglobulinemia (IgG below 400 mg/dL). Finally, our protocol includes serial monitoring of the effect of felzartamab on the overall load of immunosuppression, evaluating the load of TTV, earlier found to be associated with infection risk (and inversely rejection) [36].

A major limitation of the trial, which is not powered to detect meaningful effects of treatment versus placebo on clinical endpoints, is its small sample size. To minimize relevant imbalances between trial arms, patients will undergo stratified randomization to reduce center bias and weight for ABMR phenotype (active versus chronic active ABMR). Stratification for the presence or absence of chronic injury (most prominently transplant glomerulopathy) was chosen to account for potential variability regarding the extent of chronic injury, which is well established to be a major predictor of graft outcome, critically influencing responsiveness to treatment [43]. Even with the use of surrogate endpoints, such as eGFR slope, which may effectively predict transplant survival, larger samples would be needed. For example, in a previous trial of bortezomib in late ABMR, a sample size of 44 patients was calculated to demonstrate a meaningful treatment effect [7]. Based on a modeling exercise specifically including recipients with chronic ABMR, a currently ongoing trial evaluating clazakizumab in chronic ABMR will include 200 patients to detect relevant differences (ClinicalTrials.gov identifier: NCT03744910). The primary goal of our secondary endpoint analysis will be to get the first impression of the effect size and variability of parameters we can expect. For example, in a recent small pilot trial performed to evaluate clazakizumab in late ABMR, a significant association with eGFR slope was observed as early as 3 months after treatment initiation, even though the trial, which included 20 recipients, was not powered to detect differences in clinical endpoints [11]. Moreover, analysis of 12-month biopsies in this trial suggested modulation of molecular rejection activity, in addition to HLA antibody reduction. In this trial, we expect that the analysis of a set of efficacy endpoints, including surrogate endpoints that might predict long-term outcomes, such as eGFR slope and iBOX prediction score [27, 28], may provide a useful basis for the design of future larger trials.

Under-immunosuppression, frequently a result of medication non-adherence, may be a major trigger of de novo DSA formation and ABMR occurrence, particularly in the context of high levels of tissue incompatibility [44, 45]. One may speculate that insufficient levels of baseline immunosuppression may also promote the progression of an ongoing ABMR process, which may be of particular relevance for patients allocated to placebo treatment. Hence, as in previous trials, the design of our planned trial includes an increase in the level of baseline immunosuppression in both treatment arms (triple therapy in all included subjects; increased calcineurin inhibitor through levels), which is in line with current recommendations for the treatment of chronic ABMR [6].

If the planned trial shows a manageable safety profile and tolerability and that the results of the secondary endpoint analysis are suggestive of favorable immunological effects, our results would provide an important foundation for the conception of a larger long-duration trial powered to clarify the effect of this compound on clinically meaningful endpoints. In addition to safety data and results regarding biomarkers, renal function, and biopsies, data on PK and PD may thus help define the optimal dose and dosing intervals. The design and completion of a pivotal trial in chronic ABMR is a major challenge, given the large sample size and the extended follow-up needed to demonstrate meaningful effects on graft survival (or surrogate endpoints, such as eGFR decline or the results of follow-up biopsies).

## Trial status

Protocol version 2.0, April 30.2021. The participants’ recruitment has started in October 2021. We expect to complete the trial in April 2024.

## Supplementary Information

**Additional file 1:** Informed consent form (Felzartamab in late ABMR; Version 3.0, May 10, 2021).

**Additional file 2:** SPIRIT Checklist.

## Data Availability

The protocol of the trial has been reported in accordance with the Standard Protocol Items: Recommendations for Clinical Interventional Trials (SPIRIT) guidelines (Additional file 2). The study datasets generated and/or analyzed during the trial are available from the corresponding authors on reasonable request. Any information shared will be blinded to any identifying participant information. The results of the study are planned to be published in international peer-reviewed journals.

## Abbreviations

AE: Adverse event
ABMR: Antibody-mediated rejection
ALT: Alanine aminotransferase
AST: Aspartate aminotransferase
BAFF: B cell activating factor
CKD-EPI: Chronic Kidney Disease Epidemiology Collaboration
CMV: Cytomegalovirus
CyA: Cyclosporine A
CRF: Case report form
*CXCL*: *Chemokine (C-X-C motif) ligand*
DSA: Donor-specific antibodies
DSMB: Data and safety monitoring board
EDTA: Ethylenediaminetetraacetic acid
EC-MPA: Enteric-coated mycophenolic acid
eGFR: Estimated glomerular filtration rate
ELISA: Enzyme-linked immunosorbent assay
g: Glomerulitis
GCP: Good Clinical Practice
GLP: Good Laboratory Practice
HLA: Human leukocyte antigen
HSV: Herpes simplex virus
HZV: Herpes zoster virus
ICH: International Conference on Harmonization
Ig: Immunoglobulin
IF/TA: Interstitial fibrosis/tubular atrophy
IL-6: Interleukin-6
IL-6R: Interleukin-6 receptor
IVIG: Intravenous immunoglobulin
MedDRA: Medical Directory for Regulatory Activities
MFI: Mean fluorescence intensity
MMDx: Molecular Microscope® Diagnostic System
MMF: Mycophenolate mofetil
MPA: Mycophenolic acid
mTOR: Mammalian target of rapamycin
MVI: Microvascular inflammation
NK cell: Natural killer cell
PC: Plasma cells
PCR: Polymerase chain reaction
PD: Pharmacodynamics
PK: Pharmacokinetics
ptc: Peritubular capillaritis
SAE: Serious adverse event
TCMR: T cell-mediated rejection
TTV: Torque teno virus
ULN: Upper limit of normal
