# Supplementary material for: Safety, tolerability, and efficacy of monoclonal CD38 antibody felzartamab in late antibody-mediated renal allograft rejection: study protocol for a phase 2 trial
Source: Trials. 2022 Apr 8;23:270. doi: 10.1186/s13063-022-06198-9 (PMC8990453; doi:10.1186/s13063-022-06198-9)
Supplement: Supplementary file 1 — Additional file 1: Informed consent form (Felzartamab in late ABMR; Version 3.0, May 10, 2021). [file 13063_2022_6198_MOESM1_ESM.pdf]

PatientInneninformation[1] und Einwilligungserklärung zur Teilnahme an der klinischen Prüfung

Sicherheit, Verträglichkeit und Effektivität des CD38 Antikörpers Felzartamab bei später Antikörper-vermittelter Nierentransplantat-Abstoßung – Eine Phase 2 Pilotstudie

Sehr geehrte Patientin, sehr geehrter Patient!

Wir laden Sie ein an der oben genannten klinischen Prüfung teilzunehmen. Die Aufklärung darüber erfolgt in einem ausführlichen ärztlichen Gespräch.

Ihre Teilnahme an dieser klinischen Prüfung erfolgt freiwillig. Sie können jederzeit ohne Angabe von Gründen aus der Studie ausscheiden. Die Ablehnung der Teilnahme oder ein vorzeitiges Ausscheiden aus dieser Studie hat keine nachteiligen Folgen für Ihre medizinische Betreuung.

Klinische Prüfungen sind notwendig, um verlässliche neue medizinische Forschungsergebnisse zu gewinnen. Unverzichtbare Voraussetzung für die Durchführung einer klinischen Prüfung ist jedoch, dass Sie Ihr Einverständnis zur Teilnahme an dieser klinischen Prüfung schriftlich erklären. Bitte lesen Sie den folgenden Text als Ergänzung zum Informationsgespräch mit Ihrem Prüfarzt sorgfältig durch und zögern Sie nicht Fragen zu stellen.

Bitte unterschreiben Sie die Einwilligungserklärung nur

- wenn Sie Art und Ablauf der klinischen Prüfung vollständig verstanden haben,
- wenn Sie bereit sind, der Teilnahme zuzustimmen und
- wenn Sie sich über Ihre Rechte als Teilnehmer an dieser klinischen Prüfung im Klaren sind.

Zu dieser klinischen Prüfung, sowie zur Patienteninformation und Einwilligungserklärung wurde von der zuständigen Ethikkommission eine befürwortende Stellungnahme abgegeben.

1. Was ist der Zweck der klinischen Prüfung?

In den letzten Jahren hat sich gezeigt, dass Immunreaktionen gegen Organtransplantate (Abstoßungen) nicht nur früh nach Transplantation eine Verschlechterung der Nierenfunktion, bis hin zum Organverlust, bedingen können, sondern auch Hauptursache für einen Verlust von Nierentransplantaten im Langzeitverlauf darstellen. Hierbei spielt vor allem die Bildung von Abwehreiweißstoffen (sog. Antikörpern) gegen das (fremde) Gewebe des transplantierten Organs eine bedeutende Rolle. Eine Vielzahl von Studien hat gezeigt, dass solche Antikörper Jahre nach Transplantation das Gewebe der transplantierten Niere langsam schädigen, so dessen Funktion negativ beeinflussen, und letztlich sogar einen so schweren Gewebsschaden verursachen können, dass das Organ verloren geht.

Der Zweck dieser klinischen Prüfung ist die Erforschung der Wirksamkeit und Sicherheit von Felzartamab bei Patienten mit einer späten Antikörper-vermittelten Abstoßung nach Nierentransplantation. Dieses Medikament wurde bereits

[1] Wegen der besseren Lesbarkeit wird im weiteren Text zum Teil auf die gleichzeitige Verwendung weiblicher und männlicher Personenbegriffe verzichtet. Gemeint und angesprochen sind – sofern zutreffend – immer beide Geschlechter.

eingehend in der Behandlung des Multiplen Myeloms einer Krebserkrankung des Knochenmarks, untersucht. Felzartamab ist ein spezifischer Antikörper gegen das Oberflächen-Eiweißmolekül (sog. CD38) auf spezialisierten Immunzellen (den sogenannten Plasmazellen), die für die Bildung von schädigenden Antikörpern eine wichtige Rolle spielen. Zudem beeinträchtigt Felzartamab durch Bindung dieses Oberflächenmoleküls auch die sogenannten Natürlichen Killerzellen, besondere Immunzellen, die den Einfluss solcher Antikörper auf das transplantierte Gewebe verstärken. Dieser Wirkungsmechanismus könnte in der Transplantation, wo Antikörper-vermittelte Immunreaktionen zu einer Abstoßungsreaktion führen, von Vorteil sein. Es gibt nun erste Berichte, dass Antikörper gegen CD38 auch bei Empfängern von Organtransplantaten mit einer Antikörper-vermittelten Abstoßung eine günstige Wirkung auf den Krankheitsverlauf entfalten, dabei die Bildung schädigender Antikörper reduziert, die Organfunktion stabilisieren, und das Überleben von Transplantaten verbessern könnten.

Für CD38 Antikörper wurde in großen Studien bei Patienten mit Multiplem Myelom eine gute Verträglichkeit beschrieben. Eine wichtige Fragestellung dieser Untersuchung ist dennoch die Sicherheit einer längerfristigen Verabreichung dieser Therapie bei nierentransplantierten Patienten; denn bei solchen Patienten, die bereits eine dauerhafte immununterdrückende Therapie zur Vermeidung einer Abstoßungsreaktion erhalten, ist Felzartamab noch nicht verabreicht worden. Es wird auch die Menge und Verteilung des Medikaments im Körper untersucht („Pharmakokinetik“), und es werden, neben den üblichen Routine-Laborkontrollen, Blutproben entnommen um die Wirkung von Felzartamab im Körper zu untersuchen, wobei der Zusammenhang zwischen der Dosis und der Wirkung des Medikaments beobachtet werden soll (Pharmakodynamik). Weitere Proben Ihres Blutes werden zur Untersuchung von Antikörpern gegen die Spenderniere, Entzündungswerten, der Zusammensetzung von Blutzellen, und zur Kontrolle der Leber- und Nierenfunktion entnommen. Nach 24 Wochen sowie nach 12 Monaten erfolgt eine Transplantat-Biopsie, um die Wirkung von Felzartamab eingehend untersuchen zu können. All diese Untersuchungen dienen neben der Erforschung der Wirksamkeit, Aktivität und Verträglichkeit auch Ihrer Sicherheit.

Mit dieser Studie können letztlich erste Erkenntnisse gewonnen werden, inwieweit eine Behandlung mit Felzartamab den Verlauf einer durch Antikörper gegen das Spenderorgan vermittelten Abstoßung spät nach Nierentransplantation tatsächlich günstig beeinflussen kann.

2. Welche anderen Behandlungsmöglichkeiten gibt es?

Derzeit ist für keine Therapie ein günstiger Effekt auf späte (sog. chronische) Antikörper-vermittelte Abstoßung bewiesen. Eine kontrollierte Studie, die hier einen günstigen Einfluss bestimmter, das Immunsystem unterdrückender Medikamente beweisen konnte, ist bisher nicht veröffentlicht. In kleineren Studien oder Fallberichten beschriebene therapeutische Möglichkeiten umfassen Medikamente, die einen Einfluss auf sogenannte B-Zellen (Vorläuferzellen von stark Antikörper-produzierenden Plasmazellen) entfalten (z.B. der anti-B-Zell Antikörper Rituximab) bzw. Maßnahmen, die gleichzeitig an verschiedenen Stellen des Immunsystems angreifen und so auch die Antikörperproduktion beeinflussen können (z.B. hochdosiertes intravenöses Immunglobulin, Interleukin-6 oder Interleukin-6 Rezeptor Antikörper). Ähnlich wie bei Felzartamab wurden auch diese Substanzen allerdings noch nicht in großen Studien bei Antikörper-vermittelter Abstoßung geprüft. Sollten Sie an dieser Studie nicht teilnehmen, wird Ihr behandelnder Arzt Sie eingehend über alternative Behandlungen aufklären.

3. Wie läuft die klinische Prüfung ab?

Diese klinische Prüfung wird am AKH an der Univ. Klinik für Klinische Pharmakologie sowie an der Abteilung für Nephrologie und Dialyse (Univ. Klinik für Innere Medizin III) der Medizinischen Universität Wien durchgeführt, und es werden am AKH insgesamt 5-15 Personen daran teilnehmen. Zusätzlich wird ein weiteres Studienzentrum (Charité, Berlin) an der Studie teilnehmen (ebenso 5-15 Patienten). Insgesamt sollen 20 Patienten in diese Studie eingeschlossen werden. Alle Studien-Visiten werden an der Univ. Klinik für Klinische Pharmakologie erfolgen, in enger Zusammenarbeit mit der Abteilung für Nephrologie und Dialyse. Die Transplantatbiopsien werden im Rahmen eines tagesstationären Aufenthalts an der Abteilung für Nephrologie und Dialyse durchgeführt.

Vor Aufnahme in diese klinische Prüfung wird im Rahmen einer sogenannten Screening-Visite die Vorgeschichte Ihrer Krankheit erhoben, und Sie werden einer umfassenden ärztlichen Untersuchung unterzogen.

Ihre Teilnahme an dieser klinischen Prüfung wird voraussichtlich 12 Monate (52 Wochen) dauern.

Im Rahmen dieser klinischen Prüfung (erster Teil der Prüfung) wird eine 6-monatige Therapie mit Felzartamab mit einem sogenannten Placebo (Scheinarzneimittel, welches keinen Arzneistoff enthält) verglichen. Es ist vorgesehen, die Patienten, die an dieser Studie teilnehmen, nach dem Zufallsprinzip in zwei Gruppen einzuteilen (Randomisierung). Die Studie ist als doppel-blind Studie geplant. Das heißt, dass weder der Prüfarzt/die Prüfärztin noch der Patient weiß, ob das Medikament oder Placebo, eine identisch aussehende Infusion, verabreicht wird. Sollte es aber notwendig werden, kann Ihr Prüfarzt jederzeit in Erfahrung bringen, ob Sie das Medikament oder Placebo erhalten haben. Die Wahrscheinlichkeit ein Placebo zu erhalten, beträgt 50%.

Eine Reihe von Untersuchungen und Eingriffen werden im Zuge Ihrer Behandlung durchgeführt, gleichgültig, ob Sie nun an dieser klinischen Prüfung teilnehmen oder nicht. Diese werden von Ihrem Prüfarzt im Rahmen des üblichen ärztlichen Aufklärungsgespräches mit Ihnen besprochen.

Folgende Maßnahmen werden ausschließlich aus Studiengründen durchgeführt:

Wenn Sie an der Studie teilnehmen, erfolgen in regelmäßigen vorgegebenen Zeitintervallen Studienvisiten, anfangs in kürzeren Abständen (wöchentlich), dann in größeren Abständen, meist 4-wöchentlichen Zeitintervallen, die im Rahmen der ohnehin vorgesehenen ambulanten Routine- (Labor) Kontrollen vereinbart werden. Insgesamt sind über 12 Monate zumindest 17 Besuche notwendig. Bei gebärfähige Frauen wird vor Studienbeginn, dann im monatlichen Abstand während der klinischen Prüfung und schließlich nach Abschluss der Studie ein Schwangerschaftstest durchgeführt (siehe auch Punkt 11). Zu Beginn der Studie (Visite 1), nach 6 Monaten (Visite 10) und mit Ende der Studie (Visite 17) erfolgt durch Ihren Prüfarzt auch eine sogenannte physikalische Krankenuntersuchung. Hierbei handelt es sich um eine orientierende etwa 15-minütige körperliche Untersuchung, die es ermöglicht, rasch bedeutsame Krankheitszeichen zu erkennen. Die Untersuchung umfasst eine Beurteilung des Allgemein- und Ernährungszustands, eine orientierende Begutachtung von Haut, Augen, Zunge, Mund- und Rachenschleimhaut, ein Abhören und Abklopfen von Lunge, Herz, Bauch und Nierenregion, ein Abtasten der Schilddrüse und der Lymphknoten (Hals, Achsel, Leiste), sowie das Ertasten der Pulse an Armen und Beinen. Alle weiteren Maßnahmen zu den jeweiligen Visiten sind nachfolgend angeführt:

Visite 1 (Tag 0), Ort: Klinische Pharmakologie (tagesstationär)
Blutabnahme, Blutdruck-/Temperaturmessung, Harnuntersuchung, Nasenabstrich (Covid-19 Test), physikalische Krankenuntersuchung, anschließend Verabreichung von Felzartamab/Plazebo (30 min zuvor Vortherapie; nach der Infusion zwei Stunden Nachbeobachtung), 30 min nach Verabreichung nochmalige Blutabnahme

Visite 2 (Tag 7), Ort: Klinische Pharmakologie (tagesstationär)
Blutabnahme, Harnuntersuchung, Nasenabstrich (Covid-19 Test), Blutdruck-/Temperaturmessung
Verabreichung von Felzartamab/Plazebo (30 min zuvor Vortherapie; nach der Infusion zwei Stunden Nachbeobachtung), , 30 min nach Verabreichung nochmalige Blutabnahme

Visite 3 (Tag 14), Ort: Klinische Pharmakologie (ambulant)
Blutabnahme, Harnuntersuchung, Nasenabstrich (Covid-19 Test), Blutdruck-/Temperaturmessung
Verabreichung von Felzartamab/Plazebo; 30 min nach Verabreichung nochmalige Blutabnahme.

Visite 4 (Tag 21), Ort: Klinische Pharmakologie (ambulant)
Blutabnahme, Harnuntersuchung, Nasenabstrich (Covid-19 Test), Blutdruck-/Temperaturmessung
Verabreichung von Felzartamab/Plazebo

Visite 5 (nach 4 Wochen), Ort: Klinische Pharmakologie (ambulant)
Blutabnahme, Harnuntersuchung, Nasenabstrich (Covid-19 Test), Blutdruck-/Temperaturmessung
Verabreichung von Felzartamab/Plazebo

Visite 6 (nach 8 Wochen), Ort: Klinische Pharmakologie (ambulant)
Blutabnahme, Harnuntersuchung, Nasenabstrich (Covid-19 Test), Blutdruck-/Temperaturmessung
Verabreichung von Felzartamab/Plazebo

Visite 7 (nach 12 Wochen), Ort: Klinische Pharmakologie (ambulant)
Blutabnahme, Harnuntersuchung, Nasenabstrich (Covid-19 Test), Blutdruck-/Temperaturmessung

Verabreichung von Felzartamab/Plazebo

Visite 8 (nach 16 Wochen), Ort: Klinische Pharmakologie (ambulant)
Blutabnahme, Harnuntersuchung, Nasenabstrich (Covid-19 Test), Blutdruck-/Temperaturmessung
Verabreichung von Felzartamab/Plazebo

Visite 9 (nach 20 Wochen), Ort: Klinische Pharmakologie (ambulant)
Blutabnahme, Harnuntersuchung, Nasenabstrich (Covid-19 Test), Blutdruck-/Temperaturmessung
Verabreichung von Felzartamab/Plazebo

Visite 10 (nach 24 Wochen), Ort: Abteilung für Nephrologie und Dialyse (tagesstationär)
Blutabnahme, Harnuntersuchung, Nasenabstrich (Covid-19 Test), Blutdruck-/Temperaturmessung, EKG-Untersuchung, physikalische Krankenuntersuchung Durchführung einer Nierentransplantatbiopsie, danach 5-8 Stunden Nachbeobachtung inkl. Blutbildkontrolle (Blutabnahme) und Ultraschallkontrolle 4 Stunden nach der Biopsie

Visite 11 (nach 28 Wochen), Ort: Klinische Pharmakologie (ambulant)
Blutabnahme, Harnuntersuchung, Nasenabstrich (Covid-19 Test), Blutdruck-/Temperaturmessung

Visite 12 (nach 32 Wochen), Ort: Klinische Pharmakologie (ambulant)
Blutabnahme, Harnuntersuchung, Nasenabstrich (Covid-19 Test), Blutdruck-/Temperaturmessung

Visite 13 (nach 36 Wochen), Ort: Klinische Pharmakologie (ambulant)
Blutabnahme, Harnuntersuchung, Nasenabstrich (Covid-19 Test), Blutdruck-/Temperaturmessung

Visite 14 (nach 40 Wochen), Ort: Klinische Pharmakologie (ambulant)
Blutabnahme, Harnuntersuchung, Nasenabstrich (Covid-19 Test), Blutdruck-/Temperaturmessung

Visite 15 (nach 44 Wochen), Ort: Klinische Pharmakologie (ambulant)
Blutabnahme, Harnuntersuchung, Nasenabstrich (Covid-19 Test), Blutdruck-/Temperaturmessung

Visite 16 (nach 48 Wochen), Ort: Klinische Pharmakologie (ambulant)
Blutabnahme, Harnuntersuchung, Nasenabstrich (Covid-19 Test), Blutdruck-/Temperaturmessung

Visite 17 (nach 52 Wochen), Ort: Abteilung für Nephrologie und Dialyse (tagesstationär)
Blutabnahme, Harnuntersuchung, Nasenabstrich (Covid-19 Test), Blutdruck-/Temperaturmessung, EKG, physikalische Krankenuntersuchung Durchführung einer Nierentransplantatbiopsie, danach 5-8 Stunden Nachbeobachtung inkl. Blutbildkontrolle (Blutabnahme) und Ultraschallkontrolle 4 Stunden nach der Biopsie

Die Einhaltung der Besuchstermine, einschließlich der Anweisungen des Prüfarztes ist von entscheidender Bedeutung für den Erfolg dieser klinischen Prüfung. Bei jeder Visite erfolgen Blutabnahmen mit Kontrolle von Nieren- und Leberwerten sowie eine Bestimmung des Entzündungswerts (klinische Chemie), des Weiteren eine Kontrolle von Blutgerinnung und Blutbild, Untersuchungen, wie sie bei Routinekontrollen nach Transplantation prinzipiell vorgesehen sind. Zu allen oben angegebenen Zeitpunkten erfolgt auch, wie in der Transplantationsnachsorge üblich, eine Harnuntersuchung (Messung der Proteinausscheidung), Blutdruck- und Temperaturmessung und ärztlicher Befundbesprechung mit Besprechung der Medikamententherapie.

Zusätzlich zu den Routinekontrollen wird zudem Blut und Harn für Studienzwecke abgenommen. Vor Einschluss in die Studie erfolgt im Rahmen des sog. Screening eine Abnahme von Blut, um eine aktive Infektion auszuschließen (15 mL Blut). Zu Studienbeginn (Tag 0, sowie nach 1, 2, 4, 12 und 24 Wochen bzw. 12 Monaten erfolgt zusätzlich zu den oben genannten (Routine-) Laboruntersuchungen eine Blutabnahme (zu vier Zeitpunkten 35 mL, ansonsten 10 mL Blut) für eine genaue Bestimmung von Antikörpern gegen die transplantierte Niere, sowie von bestimmten Immunwerten aus dem Blut (z.B. immunologische Botenstoffe, Zell-Zusammensetzung und Zellfunktion). Zusätzlich erfolgt bei den ersten 3 Visiten eine Abnahme von 10 mL Blut (5 mL vor und 5 mL 30 min nach der Infusion von Felzartamab bzw. Placebo), bei den nachfolgenden Visiten von 5 mL Blut für die Bestimmung der Spiegel von Felzartamab (sowie der Bestimmung einer Immunreaktion bzw. von Antikörpern gegen Felzartamab), und darüber hinaus alle 4 Wochen die Abnahme von 10 mL Blut, um das Auftreten von Virusinfektionen auszuschließen und um regelmäßig die Immunabwehr zu prüfen (Immunglobulin-Messung). Ebenso wird, zusätzlich zu den Rotine Harnuntersuchungen, am ersten Tag, sowie nach 12, 24

und 52 Wochen Harn für die Bestimmung von bestimmten Immunwerten untersucht, um den Verlauf der Abstoßung zu beurteilen.

Sie wurden bereits bei einer vorangegangenen Routine-Kontrolle Transplantat-biopsiert und eine Abstoßung wurde nachgewiesen. Nach 24 Wochen und nach 52 Wochen (letzte Studienvisite) erfolgt eine Biopsie des Nierentransplantats, um den Einfluss von Felzartamab auf Abstoßungsprozesse und die Struktur des Transplantats genauer untersuchen zu können. Sie werden hierzu gebeten, in das Krankenhaus zu kommen. Die Biopsie wird tagesstationär (Tagesstation der Abteilung für Nephrologie und Dialyse, Innere Medizin III) im Rahmen eines Klinikaufenthalts von etwa 5-8 Stunden durchgeführt. Die Biopsie erfolgt als Stanzbiopsie Ultraschall-gezielt in lokaler Vereisung. Es werden ein bis zwei Gewebezylinder entnommen. Danach wird für 4 Stunden ein Druckverband angelegt (Bettruhe). Nach 5-8 Stunden können Sie bei komplikationslosem Verlauf und bei stabilem Blutbild (Kontrolle nach 4 Stunden) entlassen werden.

Darüber hinaus wird vor Einschluss in die Studie und vor jeder Felzartamab/Placebo-Infusion ein Covid-19-Test (Nasenabstrich) durchgeführt, um sicherzustellen, dass das Ergebnis negativ ist, bevor Sie in die Studie aufgenommen werden bzw. vor jeder Infusion von Felzartamab/Placebo. Um eine mögliche Infektion mit SARS-CoV-2 festzustellen, wird dabei ein PCR-Test oder Ag-Schnelltest durchgeführt. Für diesen Test wird ein Abstrich aus dem tiefen Nasenrachenraum genommen. Sie werden über das Ergebnis des Tests informiert. Ein negatives Testergebnis ist Voraussetzung für den Einschluss an der Klinischen Studie. Im Falle eines positiven Tests erfolgt entsprechend den gesetzlichen Vorgaben automatisch eine Meldung an die MA15, der Einschluss bzw. bei Studieninklusion Fortführung der Therapie ist bis zu einem wiederholt negativen Testergebnis nicht möglich.

4. Was ist Felzartamab?

Felzartamab ist ein Arzneimittel, welches von der Firma MorphoSys AG für den Einsatz bei Multiplem Myelom und bestimmten Autoimmunerkrankungen entwickelt wird, sich derzeit in klinischer Erprobung befindet und noch nicht zugelassen ist. Es wurde bisher bei über 100 Patienten angewendet.

Die Dosis von Felzartamab beträgt 16 mg/kg Körpergewicht pro Verabreichung, und Sie erhalten das Medikament zweimal im 2 Wochen-Abstand, dann alle 4 Wochen als Infusion über einen venösen Zugang (Venflon) (insgesamt 7 Infusionen über die ersten 6 Monate der insgesamt über 12 Monate laufenden Studie). Falls Sie der Placebogruppe zugeteilt werden, werden Sie Placebo als Infusion in den gleichen Intervallen erhalten.

5. Worin liegt der Nutzen einer Teilnahme an der Klinischen Prüfung?

Mit der Anwendung von Felzartamab kann möglicherweise eine Organschädigung durch späte Abstoßung aufgehalten werden. Dies könnte bedeuten, dass das Überleben des transplantierten Organs (Zeit bis zur Dialyse) verlängert werden kann. Es ist jedoch auch möglich, dass Sie durch Ihre Teilnahme an dieser klinischen Prüfung keinen direkten Nutzen für Ihre Gesundheit ziehen.

Die Ergebnisse dieser klinischen Prüfung sollen dazu beitragen, dass für andere Patienten, die dieselbe Erkrankung haben wie Sie, eine Behandlung gefunden wird. Gelingt es, eine gute Verträglichkeit sowie einen positiven Einfluss von Felzartamab auf den Antikörper-vermittelten Abstoßungsvorgang zu erreichen, dann hätte das Ergebnis dieser Studie große Bedeutung für die Betreuung nierentransplantierter Patienten. Darüber hinaus könnte dies auch eine Bedeutung für die Betreuung von Empfängern anderer Organtransplantate, wie Herz-, Leber-, Lungen- oder Pankreas-Transplantate, haben.

Auch wenn Sie der Placebogruppe zugeteilt werden, kann sich daraus ein Nutzen ergeben. Dieser besteht in der im Rahmen der Studie engmaschigen Überwachung der Abstoßung und der genauen Beurteilung des Verlaufs (Biopsien). Insbesondere erfolgt eine sorgfältige Anpassung der Immunsuppression, mit dem Ziel einer Optimierung der Medikamentendosis und –spiegel. Dies könnte auch ohne zusätzliche Therapie mit Felzartamab zu einer Stabilisierung des Verlaufs führen.

6. Gibt es Risiken, Beschwerden und Begleiterscheinungen?

Felzartamab kann verschiedene Nebenwirkungen und Beschwerden verursachen:

In Auswertung einer Studie zur Behandlung des Multiplen Myeloms wurden das Auftreten verschiedener Nebenwirkungen und deren Häufigkeit genau beschrieben, wobei bei diesen Patienten/Patientinnen auch ein Einfluss zusätzlicher Behandlungen und der Grunderkrankung angenommen werden muss. Für Transplantierten gibt es allerdings noch keine Ergebnisse zur Häufigkeit bestimmter Nebenwirkungen. Nachfolgend sind die wesentlichen Nebenwirkungen aus der oben genannten Studie angeführt, mit Angabe ihrer Häufigkeit (Prozentsätze für jene Patienten, die mit Felzartamab als Monotherapie oder in Kombination mit einer Steroidbehandlung behandelt wurden):

Oft kommt es zu einer meist mild ausgeprägten Infusionsreaktion, wie Schüttelfrost, Fieber, Herzklopfen oder allergieartigen Beschwerden, dann in der Regel nach der ersten Infusion (bei den nachfolgenden Verabreichungen tritt diese Nebenwirkung nur noch selten auf). Bei Myelom-Patienten, die Felzartamab als Monotherapie oder zusammen mit einem Steroid, erhielten, kam es bei etwa 20% der behandelten Patienten zu so einer Reaktion. Um das Risiko dieser Nebenwirkung zu minimieren sollen daher die ersten beiden Infusionen über 90 Minuten verabreicht werden, und erst später die Infusionsdauer verkürzt werden (auf maximal eine halbe Stunde). Zudem soll vor den ersten beiden Infusionen mit Felzartamab eine sogenannte Vortherapie erfolgen. Es ist auch möglich, dass die laufende Immunsuppression nach Nierentransplantation zusätzlich das Auftreten von Infusionsreaktionen mindert.

Infektionen: Bei Patienten mit Multiplem Myelom wurde unter einer Therapie mit CD38 Antikörpern, im Vergleich zu Placebo-behandelten Patienten, keine wesentlich erhöhtes Infektionsrisiko beschrieben. In der oben genannten Studie von Felzartamab bei Patienten/Patientinnen mit Myelom) wurden bei Behandlung mit Felzartamab allein oder in Kombination mit einer Steroidtherapie einige Infektionen, wie leichte Atemwegsinfekte oder Harnwegsinfekte beschrieben. Schwere Infektionen, wie eine Lungenentzündung oder eine schwere Bronchitis sind bei etwa 5-10% der Patienten aufgetreten. Es ist derzeit nicht bekannt, ob durch Felzartamab bei laufender immunsuppressiver Therapie nach Transplantation die Infektanfälligkeit erhöht sein kann. Entsprechend erfolgt vor Einschluss in die Studie eine sorgfältige Untersuchung, vor allem auch der Ausschluss von Virusinfektion oder Infektionen mit Bakterien und Pilzen. Teil der Voruntersuchung ist auch der Ausschluss einer Tuberkuloseerkrankung (Bluttest). Während der Behandlung wird regelmäßig der Spiegel schützender Abwehreiweißstoffe (Immunglobulin) gemessen, und gegebenenfalls schützendes Immunglobulin als Schutz infundiert.

Veränderungen des Blutbildes. Häufig kommt es zu einem meist milden bis mäßigen Abfall der Zahl weißer Blutkörperchen (dies wurde für etwa 40% der Patienten, die mit Felzartamab als Monotherapie oder in Kombination mit einer Steroidbehandlung behandelt wurden, berichtet) bzw. bei manchen Patienten ein milder Abfall der Blutplättchen (etwa 20% der Patienten). Eine Verschlechterung des roten Blutbilds (Hämoglobin), ebenso meistens mild ausgeprägt, fand sich bei etwa 40% der Patienten. Diese Veränderungen fallen bei der Laboruntersuchung auf, haben aber in aller Regel keine klinische Bedeutung. Anzumerken ist, dass diese Ergebnisse aus einer Studie bei Patienten mit einer Knochenmarkserkrankung, also der Blutbildung, gewonnen wurden.

Gelegentlich kommt es (unter felzartamab mit oder ohne Steroidtherapie) zu maximal mäßigen Beschwerden des Magen-Darmtrakts, wie Durchfällen (ca. 25%), Verstopfung (etwa 8%) oder Übelkeit (20%). Es können auch allgemeine Beschwerden, wie Müdigkeit (etwa 30%), Fieber (etwa 15%) oder Schlafstörungen (etwa 15%) auftreten. Auch wurden unter Felzartamab Kopfschmerzen (etwa 20%), oder Schwindel (etwa 10%) beschrieben. Selten ist ein milder bis mäßiger Anstieg der Leberwerte (<5% der behandelten Patienten/Patientinnen) bzw. Herzbeschwerden; hier am häufigsten ein beschleunigter Herzschlag (etwa 15%).

Vortherapie vor den ersten beiden Felzartamab-Infusionen:

Eine halbe Stunde vor den ersten beiden geplanten Infusionen mit Felzartamab (Erste Visite und nach 1 Woche) wird eine begleitende Behandlung mit einem Kortikosteroid (Prednisolon), einem Antihistaminikum (Diphenhydramin) und einem schmerzlindernden bzw. fiebersenkenden Medikaments (Paracetamol), jeweils in 100 mL, verabreicht, um möglichen Unverträglichkeitsreaktionen, die bei den ersten Infusionen von Felzartamab auftreten können, vorzubeugen. Sind Sie der Placebogruppe zugeteilt, dann wird anstelle dieser Vortherapie Placebo (3 x 100 mL Kochsalzlösung ohne Medikamente) verabreicht. Für die einzelnen Medikamente, die in dieser Vortherapie enthalten sind, sind folgende Nebenwirkungen beschrieben:

Diphenhydramin (Handelsname: Dibondrin)
Die Verabreichte einmalige Dosis von Dibondrin ist niedrig (30 mg) und liegt weit unter der erlaubten Tageshöchstdosis. Für Dibondrin sind folgende Nebenwirkungen beschrieben: Sehr häufig verursacht Dibondrin Müdigkeit (10% oder mehr). Gelegentlich (1:1000 bis 1:100) kann es zu Kreislaufproblemen, Schwindel, Benommenheit, Kopfschmerzen, Konzentrations- und Koordinationsstörungen, Sehstörungen, einer Erhöhung des Augeninnendrucks, Eindickung des Bronchialsekrets, einem Spannungsgefühl in der Brust, Magen- Darmbeschwerden, einer Störung der Blasenentleerung, einer Trockenheit von Mund, Nase und Rachen, oder Muskelschwäche kommen. Selten (1:1000 bis 1:10000) sind Herzrasen, allergische Hautreaktionen, Hautreizungen und Lichtempfindlichkeit der Haut. Sehr selten (unter 1:10000) sind Blutbildveränderungen.

Paracetamol (Handelsname: Paracetamol)
Die einmalige Verabreichung von Paracetamol in der geplanten Dosierung (1000 mg) führt selten zu Nebenwirkungen. Es kann selten (1:1000-1:10000) zu einem Unwohlsein und einem Anstieg der Leberwerte, und sehr selten (unter 1:10000) zu Blutbildveränderungen, allergischen Reaktionen der Haut, einer Verkrampfung der Luftwege und Luftnot, oder extrem selten zu einem Kreislaufschock kommen.

Prednisolon (Handelsname: Solu-Dacortin)
Eine kurzfristige Gabe ist in der Regel unproblematisch und ruft meistens gar keine oder nur geringe Nebenwirkungen hervor. Von der vorgesehenen zweimaligen Verabreichung von Prednisolon als Vortherapie (je 100 mg im Abstand von 14 Tagen) sind daher nicht die für eine Langzeittherapie bekannten typischen Nebenwirkungen, wie erhöhte Infektionsanfälligkeit, Osteoporose, Gewichtszunahme, Hautveränderungen, Augenschäden, oder Erkrankungen des Magendarmtrakts zu erwarten. Eine kurzfristige intravenöse Gabe kann aber gelegentlich zu einem Anstieg des Blutzuckers, einer Verschlechterung des Blutdrucks oder zu psychischen Veränderungen, wie zum Beispiel Unruhe oder Schlafstörungen führen. Äußerst selten sind allergische Reaktionen.

<u>Risiken einer Transplantatbiopsie:</u>

Die Transplantatbiopsien erfolgen in lokaler Vereisung im Rahmen einer tagesstationären Aufnahme. Die Biopsie wird mit Ultraschall gesteuert. Bei Ausschluss einer Gerinnungsstörung sowie unter vorübergehender Pausierung von Medikamenten, die die Blutgerinnung oder die Funktion der Blutplättchen hemmen, ist die Biopsie ein risikoarmer Eingriff. Schwere Komplikationen sind außerordentlich selten. Mögliche Risiken sind das seltene Auftreten eines Blutergusses an der Einstichstelle, der in den meisten Fällen konservativ (ohne Operation) behandelt wird. In so einem Fall ist eine Verlängerung des stationären Aufenthalts nötig (Übernahme auf eine Bettenstation). Kleinere Blutungen in die Nierenkapsel können gelegentlich Schmerzen an der Punktionsstelle verursachen. Die Notwendigkeit einer Bluttransfusion oder sogar einer Operation aufgrund einer Nachblutung ist äußerst selten. Extrem selten ist der Verlust des Organs. Es kann zudem in manchen Fällen zu einem Austreten von Blut in den Harn kommen, eine meist vorübergehende Komplikation, die in manchen Fällen, um Koagel-Bildung in der Harnblase zu verhindern, die Anlage eines Harnkatheters erfordert. Eine seltene Komplikation ist das Auftreten einer sogenannten arterio-venösen Fistel (Verbindung einer Arterie mit einer Vene im Transplantat), welche dann manchmal eine Organfunktions-Verschlechterung, die dann eine besondere Behandlung notwendig macht (Verschließen der Fistel im Rahmen eines radiologischen Eingriffs), verursachen kann. Eine weitere außerordentlich seltene Komplikation ist die Verletzung benachbarter innerer Organe (Leber, Milz). Extrem selten tritt eine allergische Reaktion gegen das verabreichte Lokalanästhetikum auf. Dies kann zu Juckreiz, Niesen, Hautauschlag, Schwindel und Erbrechen führen. Schwerwiegende Komplikationen im Bereich lebenswichtiger Funktionen (Herz, Kreislauf, Atmung) sind hier allerdings extrem selten.

Darüber hinaus sind für die Studie wiederholt Blutabnahmen und Venenpunktionen (Venflon) für diagnostische Maßnahmen notwendig (ein Teil der Blutabnahmen erfolgt allerdings im Rahmen von Routinekontrollen in der Ambulanz).

<u>Venenpunktionen können folgende Beschwerden und Komplikationen verursachen:</u>

Es erfolgt bei jeder Visite eine Venenpunktion und Blutabnahme (an Infusionstagen wird eine Verweilkanüle/Venflon gelegt). Gelegentlich verursacht eine Venenpunktion länger anhaltende Schmerzen an der Einstichstelle oder lokal ein Hämatom (blauer Fleck). Sehr selten ist eine Infektion im Bereich der Einstichstelle (ev. Notwendigkeit einer antibiotischen Therapie) oder eine Venenentzündung, gelegentlich mit anhaltender Schädigung des Gefäßes. Verletzungen von Nerven sind außerordentlich selten.

<u>Nasenabstrich</u>

Für den Nasenabstrich wird mit einem Wattestieltupfer zuerst von einer, dann von der anderen Nasenöffnung (Nasopharynxraum) Nasensekret von der Schleimhaut abgestrichen. Die Berührung der hinteren Rachenwand mit dem Wattebausch kann einen Würgereflex auslösen. Beim Abstreichen der Nasenöffnung kann es zu minimalen Schleimhautverletzungen (Nasenbluten) kommen.

7. Zusätzliche Einnahme von Arzneimitteln?

Bei Nachweis einer späten Antikörper-vermittelten Abstoßung muss eine zu gering dosierte begleitende Immunsuppression vermieden werden. Dies gilt vor allem für den Fall, dass bei Ihnen die dauerhaft einzunehmende, das Immunsystem unterdrückende Therapie (Immunsuppression) bereits auf eine sehr niedrige Dosis reduziert wurde. Dies betrifft vor allem folgende Fälle:

Ihre Immunsuppression beinhaltet kein Kortison: In diesem Fall wird eine Kortison-Therapie mit niedriger Dosis begonnen (Aprednislon® mit 5 mg/Tag).

Ihre Immunsuppression beinhaltet keinen Proliferationshemmer (Mycophenolsäure oder Azathioprin): In diesem Fall wird eine Therapie mit dem Medikament Mycophenolsäure (z.B. CellCept® oder Myfortic®) begonnen, anfangs in niedriger Dosis. Wenn Sie Mycophenolsäure gut vertragen, wird dann schrittweise auf maximal 2 x 1000 mg (CellCept®) bzw, 2x720 mg (Myfortic®) erhöht.

8. Hat die Teilnahme an der klinischen Prüfung sonstige Auswirkungen auf die Lebensführung und welche Verpflichtungen ergeben sich daraus?

Die Teilnahme an der Prüfung bedingt zusätzliche Ambulanzbesuche für die Verabreichung der Medikation bzw. Placebo. Darüber hinaus sind regelmäßige ambulante Kontrollen, die auch notwendige klinische Routinekontrollen einbeziehen vorgesehen. Ansonsten sind keine Auswirkungen auf die Lebensführung bzw. Verpflichtungen zu erwarten.

9. Was ist zu tun beim Auftreten von Symptomen, Begleiterscheinungen und/oder Verletzungen?

Sollten im Verlauf der klinischen Prüfung irgendwelche Symptome, Begleiterscheinungen oder Verletzungen auftreten, müssen Sie diese Ihrem Prüfarzt mitteilen, bei schwerwiegenden Begleiterscheinungen umgehend, ggf. telefonisch (Telefonnummern, etc. siehe unten).

10. Versicherung

Als Teilnehmer an dieser klinischen Prüfung besteht für Sie der gesetzlich vorgeschriebene verschuldensunabhängige Versicherungsschutz (Personenschadenversicherung gemäß § 32 Arzneimittelgesetz/§ 47 Medizinproduktegesetz, der alle Schäden abdeckt, die an Ihrem Leben oder Ihrer Gesundheit durch die an Ihnen durchgeführten Maßnahmen der klinischen Prüfung verursacht werden können, mit Ausnahme von Schäden auf Grund von Veränderungen des Erbmaterials in Zellen der Keimbahn.

Die Versicherung wurde für Sie bei der Zürich Versicherungs-Aktiengesellschaft (A-1010 Wien, Schwarzenbergplatz 15, Österreich, +43 1 50125-1338 (Telefon), +43 1 50125 1507 (Fax), http://www.zurich.at), unter der Polizzennummer 07229622-2 (Rahmenvertrag der MedUniWien) abgeschlossen. Auf Wunsch können Sie in die Versicherungsunterlagen Einsicht nehmen.

Im Schadensfall können Sie sich direkt an den Versicherer wenden und Ihre Ansprüche selbständig geltend machen. Für den Versicherungsvertrag ist österreichisches Recht anwendbar, die Versicherungsansprüche sind in Österreich einklagbar.

Zur Unterstützung können Sie sich auch an die Patientenanwaltschaft, Patientenvertretung oder Patientenombudsschaft wenden.

Um den Versicherungsschutz nicht zu gefährden

- dürfen Sie sich während der Dauer der klinischen Prüfung einer anderen medizinischen Behandlung nur im Einvernehmen mit Ihrem behandelnden Prüfarzt unterziehen **(ausgenommen davon sind Notfälle).** Dies gilt auch für die zusätzliche Einnahme von Medikamenten oder die Teilnahme an einer anderen Studie.
- müssen Sie dem behandelnden Prüfarzt - oder der oben genannten Versicherungsgesellschaft - eine Gesundheitsschädigung, die als Folge der klinischen Prüfung eingetreten sein könnte, unverzüglich mitteilen.
- müssen Sie alles Zumutbare tun um Ursache, Hergang und Folgen des Versicherungsfalles aufzuklären und den entstandenen Schaden gering zu halten. Dazu gehört ggf. auch, dass Sie Ihre behandelnden Ärzte ermächtigen, vom Versicherer geforderte Auskünfte zu erteilen.

11. Informationen für gebärfähige Frauen – Schwangerschaftstest

Schwangere und stillende Frauen dürfen an dieser klinischen Prüfung NICHT teilnehmen. Ihr Prüfarzt wird bei prinzipieller Möglichkeit einer Schwangerschaft bei Ihnen oder bei Ihrer Partnerin, mit Ihnen zuverlässige Verhütungsmethoden im Detail besprechen und Sie (bzw. Ihre Partnerin) anhalten, diese bis 5 Monate nach Beendigung der Studie fortzuführen.

Als gebärfähige Frau dürfen Sie an der klinischen Prüfung nur teilnehmen,

- wenn ein Arzt vor und monatlich während der klinischen Prüfung das Nichtvorliegen einer Schwangerschaft (Schwangerschaftstest) feststellt. Es wird Ihnen weiters die Durchführung eines Schwangerschaftstests nach Abschluss der Studie empfohlen.
- wenn Sie sich verpflichten während der Dauer eine zuverlässige Art der Empfängnisverhütung (Pille, Spirale) zu praktizieren.

Sollten Sie (oder Ihre Partnerin) dennoch während der klinischen Prüfung schwanger werden oder den Verdacht haben, dass Sie schwanger geworden sind, informieren Sie bitte umgehend Ihren Prüfarzt.

Bislang gibt es keine Informationen zu Schwangerschaften unter einer Therapie mit Felzartamab.

12. Wann wird die klinische Prüfung vorzeitig beendet?

Sie können jederzeit auch ohne Angabe von Gründen, Ihre Teilnahmebereitschaft widerrufen und aus der klinischen Prüfung ausscheiden ohne dass Ihnen dadurch irgendwelche Nachteile für Ihre weitere medizinische Betreuung entstehen.

Ihr Prüfarzt wird Sie über alle neuen Erkenntnisse, die in Bezug auf diese klinische Prüfung bekannt werden, und für Sie wesentlich werden könnten, umgehend informieren. Auf dieser Basis können Sie dann Ihre Entscheidung zur **weiteren** Teilnahme an dieser klinischen Prüfung neu überdenken.

Es ist aber auch möglich, dass Ihr Prüfarzt entscheidet, Ihre Teilnahme an der klinischen Prüfung vorzeitig zu beenden, ohne vorher Ihr Einverständnis einzuholen. Die Gründe hierfür können sein:

a) Sie können den Erfordernissen der Klinischen Prüfung nicht entsprechen;

b) Ihr Prüfarzt hat den Eindruck, dass eine weitere Teilnahme an der klinischen Prüfung nicht in Ihrem Interesse ist

Sofern Sie sich dazu entschließen, vorzeitig aus der klinischen Prüfung auszuscheiden, oder Ihre Teilnahme aus einem der oben genannten Gründe vorzeitig beendet wird, ist es für Ihre eigene Sicherheit wichtig, dass Sie sich einer normalen Kontrolluntersuchung unterziehen. Diese besteht meistens aus einer körperlichen Untersuchung sowie aus Laboruntersuchungen.

13. Datenschutz

Im Rahmen dieser klinischen Prüfung werden Daten über Sie erhoben und verarbeitet. Es ist grundsätzlich zu unterscheiden zwischen

1) jenen personenbezogenen Daten, anhand derer eine Person direkt identifizierbar ist (z.B. Name, Geburtsdatum, Adresse, Sozialversicherungsnummer, Bildaufnahmen...),
2) pseudonymisierten personenbezogenen Daten, das sind Daten, bei denen alle Informationen, die direkte Rückschlüsse auf die konkrete Person zulassen, entweder entfernt, durch einen Code (z. B. eine Zahl) ersetzt oder (z.B. im Fall von Bildaufnahmen) unkenntlich gemacht werden. Es kann jedoch trotz Einhaltung dieser Maßnahmen nicht vollkommen ausgeschlossen werden, dass es unzulässigerweise zu einer Re-Identifizierung kommt.
3) anonymisierten Daten, bei denen eine Rückführung auf die konkrete Person ausgeschlossen werden kann.

Zugang zu den Daten, anhand derer Sie direkt identifizierbar sind (siehe Punkt 1), haben der Prüfarzt und andere Mitarbeiter des Prüfzentrums, die an der klinischen Prüfung oder Ihrer medizinischen Versorgung mitwirken. Zusätzlich können autorisierte und zur Verschwiegenheit verpflichtete Beauftragte des Sponsors, der Medizinischen Universität Wien, sowie Beauftragte von in- und/ oder ausländischen Gesundheitsbehörden und jeweils zuständige Ethikkommissionen in diese Daten Einsicht nehmen, soweit dies für die Überprüfung der ordnungsgemäßen Durchführung der klinischen Prüfung notwendig ist. Sämtliche Personen, die Zugang zu diesen Daten erhalten, unterliegen im Umgang mit den Daten den jeweils geltenden nationalen Datenschutzbestimmungen und/oder der EU-Datenschutz-Grundverordnung (DSGVO).

Der Code, der eine Zuordnung der pseudonymisierten Daten zu Ihrer Person ermöglicht, wird nur an Ihrem Prüfzentrum aufbewahrt.

Eine Weitergabe der Daten erfolgt nur in pseudonymisierter oder anonymisierter Form.

Für etwaige Veröffentlichungen werden nur die pseudonymisierten oder anonymisierten Daten verwendet.

Im Rahmen dieser klinischen Prüfung ist keine Weitergabe von Daten in Länder außerhalb der EU (Drittland) vorgesehen.

Ihre Einwilligung bildet die Rechtsgrundlage für die Verarbeitung Ihrer personenbezogenen Daten. Sie können die Einwilligung zur Erhebung und Verarbeitung Ihrer Daten jederzeit ohne Begründung widerrufen. Nach Ihrem Widerruf werden keine weiteren Daten mehr über Sie erhoben. Die bis zum Widerruf erhobenen Daten können allerdings weiter im Rahmen dieser klinischen Prüfung verarbeitet werden.

Nach der DSGVO stehen Ihnen grundsätzlich die Rechte auf Auskunft, Berichtigung, Löschung, Einschränkung der Verarbeitung, Datenübertragbarkeit und Widerspruch zu, soweit dies die Ziele der klinischen Prüfung nicht unmöglich macht oder ernsthaft beeinträchtigt und soweit dem nicht andere gesetzliche Vorschriften widersprechen.

Das gemäß DSGVO vorgesehene Recht auf Löschung Ihrer im Rahmen dieser klinischen Prüfung verarbeiteten Daten steht Ihnen aufgrund von Regelungen nach dem Arzneimittelgesetz und Medizinproduktegesetz nicht zu. Zusätzlich ist bei einer klinischen Prüfung nach dem Arzneimittelgesetz das Recht auf Datenübertragbarkeit außer Kraft gesetzt.

Die voraussichtliche Dauer der klinischen Prüfung ist 30 Monate. Die Dauer der Speicherung Ihrer Daten über das Ende oder den Abbruch der klinischen Prüfung hinaus ist durch Rechtsvorschriften geregelt.

Falls Sie Fragen zum Umgang mit Ihren Daten in dieser klinischen Prüfung haben, wenden Sie sich zunächst an Ihren Prüfarzt. Dieser kann Ihr Anliegen ggf. an die Personen, die für den Datenschutz verantwortlich sind, weiterleiten.

Kontaktdaten der Datenschutzbeauftragten der an dieser klinischen Prüfung beteiligten Institutionen:

Datenschutzbeauftragte/r der MedUni Wien: datenschutz@meduniwien.ac.at

Datenschutzverantwortliche/r des AKH: datenschutz@akhwien.at

Sie haben das Recht, bei der österreichischen Datenschutzbehörde eine Beschwerde über den Umgang mit Ihren Daten einzubringen (www.dsb.gv.at; E-Mail: dsb@dsb.gv.at).

14. Entstehen für die Teilnehmer Kosten? Gibt es einen Kostenersatz oder eine Vergütung?

Durch Ihre Teilnahme an dieser klinischen Prüfung entstehen für Sie keine zusätzlichen Kosten. Eine Vergütung ist nicht vorgesehen.

15. Möglichkeit zur Diskussion weiterer Fragen

Für weitere Fragen im Zusammenhang mit dieser klinischen Prüfung stehen Ihnen Ihr Prüfarzt und seine Mitarbeiter gern zur Verfügung. Auch Fragen, die Ihre Rechte als Patient und Teilnehmer an dieser klinischen Prüfung betreffen, werden Ihnen gerne beantwortet.

Name der Kontaktperson:	aoProf. Dr. Georg Böhmig
Erreichbar unter:	01 40400 43630 oder 39304, Pager: 81-4317
Name der Kontaktperson:	aoProf. Dr. Bernd Jilma
Erreichbar unter:	01 40400 29810
Name der Kontaktperson:	Dr. Konstantin Doberer
Erreichbar unter:	01 40400 55260 oder 62070, Pager: 81-4307

In Notfällen ist stets ein Mitarbeiter der Abteilung (Univ. Klinik für Klinische Pharmakologie) über „Notfallhandy" rund um die Uhr erreichbar (Notfallhandy: +43 (0) 676/7157450).

16. Wo kann ich weitere Informationen einholen?

Sie haben zudem die Möglichkeit, bei der Patientenvertretung oder der Patientenanwaltschaft zusätzliche Informationen einzuholen. Wiener Pflege-, Patientinnen-und Patientenanwaltschaft Ramperstorffergasse 67, 1050 Wien; Tel.: +43 1 587 12 04 Fax: +43 1 586 36 99

17. Sollten andere behandelnde Ärzte von der Teilnahme an der klinischen Prüfung informiert werden?

Wenn Sie es wünschen, können Sie Ihren Hausarzt über Ihre Teilnahme an dieser klinischen Prüfung informieren. Sollte bei Ihrem Hausartzt zusätzlich eine Kontrolle oder Untersuchung erfolgen, bitten wir Sie Ihren Prüfarzt zu infomieren.

18. Einwilligungserklärung

Name des Patienten:

Geb.Datum:

Ich erkläre mich bereit, an der klinischen Prüfung „Sicherheit, Verträglichkeit und Aktivität des CD38 Antikörpers Felzartamab bei später Antikörper-mediierter Nierentransplantat-Abstoßung“teilzunehmen. Ich bin darüber aufgeklärt worden, dass ich die Teilnahme ohne nachteilige Folgen, insbesondere für meine medizinische Betreuung, ablehnen kann.

Ich bin von Frau/Herrn (Dr.med.) ....ausführlich und verständlich über die klinische Prüfung, mögliche Belastungen und Risiken, sowie über Wesen, Bedeutung und Tragweite der klinischen Prüfung, die bestehende Versicherung sowie die sich für mich daraus ergebenden Anforderungen aufgeklärt worden. Ich habe darüber hinaus den Text dieser Patientenaufklärung und Einwilligungserklärung, die insgesamt *13* Seiten umfasst, gelesen. Aufgetretene Fragen wurden mir vom Prüfarzt verständlich und zufriedenstellend beantwortet. Ich hatte ausreichend Zeit, mich zu entscheiden. Ich habe zurzeit keine weiteren Fragen mehr.

Ich werde den ärztlichen Anordnungen, die für die Durchführung der klinischen Prüfung erforderlich sind, Folge leisten, behalte mir jedoch das Recht vor, meine freiwillige Mitwirkung jederzeit zu beenden, ohne dass mir daraus Nachteile, insbesondere für meine medizinische Betreuung, entstehen.

Ich stimme ausdrücklich zu, dass meine im Rahmen dieser klinischen Prüfung erhobenen Daten wie im Abschnitt „Datenschutz“ dieses Dokuments beschrieben verarbeitet werden.

Für den Fall, dass ich aus der klinischen Prüfung ausscheide, bin ich einverstanden, dass meine Proben weiterhin aufbewahrt und analysiert werden, wie in dieser Information und – wenn zutreffend – in den Informationen zu den Substudien beschrieben:

☐ ja ☐ nein

Eine Kopie dieser Patienteninformation und Einwilligungserklärung habe ich erhalten. Das Original verbleibt beim Prüfarzt.

....
(Datum und Unterschrift des Patienten)

....
(Datum, Name und Unterschrift des verantwortlichen Prüfarztes)

(Der Patient erhält eine unterschriebene Kopie der Patienteninformation und Einwilligungserklärung, das Original verbleibt im Studienordner des Prüfarztes.)
